# Beneficiary feedback mechanisms: Exploring ways to systematically integrate community feedback in mass drug administration delivery for NTDs

**DOI:** 10.1371/journal.pntd.0014373

**Published:** 2026-06-08

**Authors:** Jake D. Mathewson, Charlie Aardewijn, Sake J. de Vlas, Dunstan J. Matungwa, Mirjam I. Bakker

**Affiliations:** 1 Department of Epidemiology, KIT Royal Tropical Institute, Amsterdam, The Netherlands; 2 Department of Public Health, Erasmus MC, University Medical Center Rotterdam, Rotterdam, The Netherlands; 3 Department of Research Coordination and Promotion, National Institute for Medical Research, Mwanza, Tanzania; University of Buea, CAMEROON

## Abstract

**Background:**

Neglected tropical diseases (NTDs) affect over one billion people globally and disproportionately impact marginalized populations in low- and middle-income countries. Mass drug administration (MDA) is the WHO recommended strategy for controlling five major NTDs. However, persistent operational challenges, like treatment fatigue, mistrust, and systematically missed populations, undermine MDA effectiveness. Beneficiary Feedback Mechanisms (BFM) are emerging tools in global health that enable structured collection of community input to improve program delivery, but their application in NTD programs remains limited and understudied. This study examines how BFM are operationalized within routine MDA programs, providing operational insight into their perceived value, practical applications, and implementation challenges across diverse stakeholder roles.

**Methods:**

This study used semi-structured key informant interviews (KII) to examine how BFM can be operationalized to enhance MDA delivery. Interviews were conducted with 14 informants involved in MDA implementation, funding, monitoring and evaluation, and research, primarily across countries in sub-Saharan Africa that were supported by the Accelerating the Sustainable Control and Elimination of Neglected tropical Diseases (ASCEND) program. Participants were selected purposively based on their professional experience with BFM and MDA. Transcripts were analyzed using a thematic analysis approach in NVivo software, applying both inductive and deductive coding strategies to identify patterns and develop meaningful themes.

**Results:**

Informants identified that BFM yielded timely and actionable feedback, particularly through daily community drug distributor meetings and post-MDA surveys, as the most impactful. Integrating BFM into existing systems was favored over new platforms, particularly in resource-constrained settings. Key barriers included limited integration into digital monitoring systems and lack of dedicated personnel to review and respond to feedback. BFM also highlighted issues that may have hindered optimal drug coverage in some communities, including gender barriers and misinformation. Informants emphasized that closing the loop, communicating back to communities how their feedback was used, was essential to maintaining trust and engagement in future MDA activities.

**Conclusions:**

BFM were perceived by informants as a promising pathway to enhance the equity, responsiveness, and effectiveness of MDA programs. Their success depends on timely use, integration into routine systems, and capacity to address community concerns. Multilateral organizations such as WHO can support scale-up by issuing guidance and standardizing tools. Further research is needed to evaluate impact and best practices for implementation.

## Author summary

Neglected tropical diseases (NTDs) affect more than one billion people, primarily in low- and middle-income countries. Five of these diseases are controlled through large-scale drug distribution campaigns, in which medicines are delivered to entire communities once or twice per year. While this strategy has led to substantial progress, reaching all eligible individuals remains a challenge, and programs often lack systematic ways to understand and respond to community concerns that can limit participation. In this study, we explored how beneficiary feedback mechanisms (BFM), structured ways of collecting and responding to community perspectives, are viewed by professionals working in drug distribution programs. We interviewed 14 individuals involved in neglected tropical disease policy, research, and program implementation across sub-Saharan Africa, including government officials, implementing partners, donors, and researchers. Professionals working across neglected tropical disease programs described beneficiary feedback as a potentially useful addition to routine drug distribution. They emphasized that feedback should be embedded within existing tools and meetings, rather than implemented as a standalone platform. Informants highlighted the importance of clear responsibility for reviewing feedback and ensuring that it leads to visible program adjustments, including closing the loop with communities. At the same time, they noted structural constraints, including limited staffing, digital systems that do not capture qualitative data, and competing priorities. These perspectives suggest that community feedback warrants thoughtful integration and formal evaluation within drug distribution programs. Empirical studies are now needed to assess whether such mechanisms improve coverage or program performance.

## Background

Neglected tropical diseases (NTDs) are a group of diseases and conditions that disproportionately affect the world’s most impoverished communities in low- and middle-income countries (LMICs) [1]. Over one billion people globally are affected by NTDs, yet because their control receives comparatively less attention, funding, and prioritization, they are termed “neglected” [1]. NTDs can lead to disability, stigma, and social and economic exclusion [1]. To reduce both transmission and the progression of devastating diseases, the World Health Organization (WHO) recommends mass drug administration (MDA) as the primary strategy for controlling five key NTDs: lymphatic filariasis, onchocerciasis, schistosomiasis, soil-transmitted helminthiases, and trachoma [2]. MDA involves distributing preventive chemotherapy (PC) to at-risk populations, regardless of individual diagnosis, and is repeated in endemic areas to maintain control and interrupt transmission [3].

Despite MDA’s proven effectiveness in reducing disease prevalence and transmission [4,5], its delivery faces persistent operational and social challenges [4,6–8]. These include ethical concerns about autonomy and program ownership [4], and barriers to community acceptance such as mistrust, fear of side effects, and treatment fatigue [9]. Any combination of these factors may contribute to sub-optimal drug coverage, where the target proportions of the community are not given medications, limiting the success of MDA in achieving disease elimination goals. It is also increasingly believed that populations are being systematically missed during MDA delivery, and that by never treating certain groups, disease transmission and the progression of severe morbidities associated with NTDs persist [10].

Improving community trust and participation is increasingly seen as critical to overcoming these barriers [11]. One approach that has gained attention in global health programming is the integration of Beneficiary Feedback Mechanisms (BFM), or structured cyclical processes for systematically collecting and responding to feedback from program recipients, and communicating the resulting changes during program delivery [12]. While there is limited evidence on the use of BFM for MDA, or even within the greater sphere of NTD control, there is a growing consensus that improving methods of community engagement may in turn play an important role in improving health outcomes [11,13,14]. In other programs within the health sector, collection of feedback has been demonstrated to be supportive in improving program quality, accountability, and community ownership [15,16]. This includes research on the importance of community feedback for vaccination campaigns, which, like MDA, rely on effectively reaching a certain proportion of the population to reduce disease transmission [17]. Evidence of their application in NTD programs, however, remains limited, with a paucity of research available to support best practices on effective implementation despite the perceived importance of engaging communities [11,18]. While informal feedback and supervision mechanisms exist within MDA campaigns, there is little evidence of standardized or scalable approaches that systematically incorporate community feedback into program design or adjustment.

**Fig 1:**
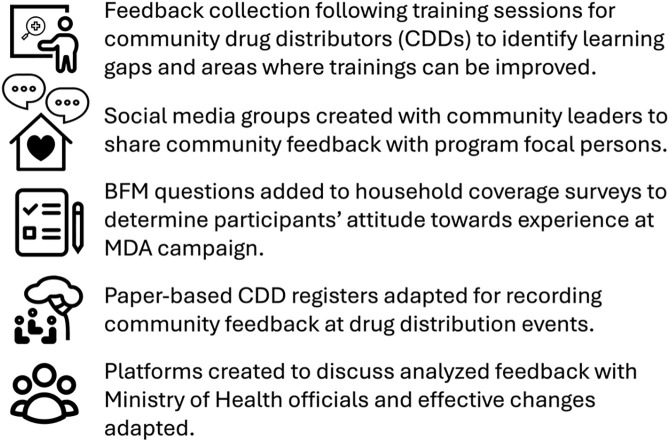
Overview of primary BFM methods used for MDA in the ASCEND project. Icons used in this figure are open-source and were obtained from Freeicons.io (Health Icons by ResolveTSL; Heroicons by Tailwind Labs) under the MIT License, using URL https://www.freeicons.org/icons/health-icons.

One of the few large-scale initiatives to explore the use of BFM in the NTD space was the Accelerating the Sustainable Control and Elimination of Neglected Tropical Diseases (ASCEND) program, funded by the UK Foreign, Commonwealth and Development Office (FCDO). Supporting MDA implementation across 24 countries across sub-Saharan Africa and southern Asia, the ASCEND program piloted several feedback approaches (Fig 1) [19,20], but systematic evidence of its impact remains scarce. FCDO defines BFM as a tool designed to gather and respond to the views of recipients of aid, often called beneficiaries [21]. While FCDO uses the term BFM, the process of collecting feedback from drug recipients surrounding MDA is also referred to as “participatory feedback” [22,23], “downward accountability mechanisms” [24], and “community feedback” [25]. In this manuscript, we use the term BFM to refer specifically to structured processes for systematically collecting, analyzing, and responding to community feedback within MDA programs.

Although WHO’s preventive chemotherapy implementation guidelines and disease specific monitoring and evaluation frameworks emphasize the importance of community engagement, social mobilization, and drug coverage validation, they stop short of prescribing concrete methods for systematically collecting, analyzing, and responding to beneficiary feedback [26]. References to supervisors’ coverage tools, coverage evaluation surveys, and data quality audits provide indirect channels through which concerns can surface, but explicit standards for BFM design, integration, or accountability loops are absent [3]. While WHO has embraced the Leave No One Behind (LNOB) strategy as part of the NTD 2030 Roadmap, practical guidance on how beneficiary feedback can be operationalized to ensure the inclusion of marginalized and systematically missed populations is not yet articulated [27]. As a result, national NTD programs often rely on ad hoc approaches or donor-driven pilots rather than clear global recommendations for embedding feedback systems into MDA.

Given the ongoing need to improve MDA performance, particularly in hard to reach and marginalized populations, there is growing recognition that integration of methods to engage communities could help programs better adapt to specific community needs, enhance trust, and ultimately improve treatment coverage [28–33]. Yet, no evidence-based strategy exists to demonstrate how to best operationalize the use of feedback, nor which tools are most effective when conducting MDA for NTDs.

This study seeks to address that gap by examining how BFM are operationalized within routine MDA programs for NTDs. Focusing specifically on the community feedback component of BFM in MDA delivery, we draw on semi-structured interviews with key informants across sub-Saharan Africa to analyze stakeholder experiences, perceived value, practical applications and implementation challenges related to BFM. By synthesizing these perspectives, the study advances understanding of how BFM function within existing MDA systems and identifies practical considerations for their systematic integration into routine program activities.

## Methods

### Ethics statement

The study was reviewed by the KIT Royal Tropical Institute Research Ethics Committee and deemed to pose minimal risk to participants, as it involved anonymized interviews with professionals discussing programmatic experiences. Consequently, the committee granted an ethical waiver (Ref no S-235). Participants received an information letter outlining the purpose of the study, voluntary participation, and confidentiality measures, and provided written informed consent prior to the interviews. No financial or material incentives were provided for participation. Consent was reconfirmed verbally at the start of each interview.

All data were anonymized and stored on a secure server in compliance with the General Data Protection Regulation (GDPR) practices. Access to the server was limited to the lead and supportive researchers, and all transcript data will be deleted 6 months after publication as outlined in the consent form. The study was conducted in line with the ethical principles of the Declaration of Helsinki.

### Study design

This study used an exploratory qualitative design to investigate how BFM can be operationalized to improve MDA programs for NTD control. Semi-structured key informant interviews (KII) were chosen as the primary data collection method to capture stakeholder experiences, operational structures, and perceptions of BFM use in sub-Saharan Africa. This design was well suited for exploring an under-researched area that required both conceptual and practical insights. KIIs provide an opportunity to collect information from a range of professionals with firsthand knowledge of the motivations, behaviors and perspectives of both the community and key stakeholders [34,35].

### Participant selection and sample size

Participants were purposively selected based on their involvement in MDA program implementation, funding, monitoring and evaluation, or research, with a specific focus on those with experience in the ASCEND-supported countries where implementation of BFM was known to have taken place. Eligible participants held roles in national NTD programs, Ministries of Health, donor agencies, non-governmental organizations (NGOs), and research institutions. The sample included representatives who had contributed to work in a variety of anglophone, lusophone, and francophone countries across sub-Saharan Africa to ensure geographical and institutional diversity. As participants were recruited largely from within our NTD research network, and as prior exposure to BFM was necessary for participation in the study, the potential for selection bias was acknowledged. Efforts were made throughout the research process, including during creation of interview questions, facilitation of interviews, and analysis, to limit the influence of this bias in the study findings. These efforts included standardized interview guides, independent double-coding, and comparison of responses across stakeholder groups, among others. As the study was conducted within ASCEND supported contexts in sub-Saharan Africa, the findings are most directly applicable to preventive chemotherapy NTD programs operating under similar implementation structures, including community-based MDA delivery and routine drug coverage monitoring. While they may be relevant to other MDA platforms, transferability to non-preventive chemotherapy NTD programs or substantially different health system contexts should be interpreted cautiously.

Thirty individuals were invited via email to participate in interviews. Fourteen consented within the two-month data collection period. No trends in roles or geographic locations were noted among non-respondents. Interviews were conducted on a rolling basis as participants consented, until all 14 had been completed. Participants represented a range of roles: funders (n = 2), NGO implementing partner staff (n = 1), monitoring, evaluation and learning (MEL) officers (n = 2), national program staff (n = 6), and researchers (n = 3). The final sample captured diverse perspectives across implementation and policy levels, as shown in Table 1. No further recruitment was pursued after the fourteenth interview given the diversity achieved across stakeholder roles, limited data collection timeframe and recurrence of themes from earlier interviews.

**Table 1:** Overview of key informants interviewed by organizational affiliation and role. Abbreviations: F = funder, IP = implementing partner, MS = monitoring, evaluation and learning staff, PI = national NTD program managers, R = professionals working in academia.

Organization	Position	Abbreviation
Funder	Analyst	F1
Funder	Fund manager	F2
NGO	Implementing partner	IP1
National NTD program - MEL staff	MEL staff member	MS1
National NTD program - MEL staff	Regional MEL Advisor	MS2
National NTD program managers	Regional manager 1	PI1
National NTD program managers	Country Lead	PI2
National NTD program managers	Country Lead	PI3
National NTD program managers	Regional manager 2	PI4
National NTD program managers	Local manager	PI5
National NTD program managers	National program staff	PI6
Academia	Researcher in public health	R1
Academia	Researcher in social change programs	R2
Academia	Researcher in the field of BFM	R3

Sample size was guided by principles of thematic saturation [36] and information power [37] rather than numerical representativeness. Preliminary assessment of thematic recurrence occurred during the data collection period, followed by a more systematic evaluation of saturation was during coding and analysis. While interviews were not fully coded concurrently with data collection, ongoing review indicated a repetition of key themes. After completion of all interviews, comprehensive coding process confirmed that no substantively new codes or thematic categories were emerging across stakeholder groups. Consistent with established operationalizations of thematic saturation [38], recruitment ceased after 14 interviews, as additional interviews reinforced previously identified themes rather than generating new insights.

Although some stakeholder categories included few participants, these individuals held roles with broad oversight or cross-country responsibilities, providing insight across multiple implementation contexts. Overall, participant selection prioritized analytic depth and information-rich informants with direct relevance to BFM implementation in ASCEND-supported settings.

### Data collection and instruments

KIIs were conducted via Microsoft Teams from April 17^th^ until May 23^rd^ 2024. Each interview lasted between 45 and 60 minutes (mean: 54 minutes). The semi-structured interview guide was based on a review of the literature and programmatic context and piloted prior to implementation. Topics included previously used feedback mechanisms, professional experience with BFM, and perceptions on best practices of integration into MDA programs. The guide was adapted iteratively to explore emerging themes raised through conversations with key informants. Further themes from the semi-structured guide are presented in *the Annex 1 Interview Guide*, which was piloted prior to the KIIs.

### Data analysis

Interview audio recordings were transcribed using Microsoft 365 Word and analyzed using NVivo 1.7.2. A hybrid thematic analysis approach, incorporating both inductive and deductive coding, was used to provide depth and breadth in the analysis and aimed to reduce the risk of confirmation bias [39]. The deductive component was informed by prior operational literature on BFM, including FCDO supported program reports, as well as established implementation frameworks such as the USAID NTD Sustainability Framework [40], which outlines key dimensions of NTD program delivery and sustainability. These sources provided sensitizing concepts that structured early coding while allowing themes to evolve inductively through iterative transcript review. Inductive coding involved detailed reading of the transcripts to derive concepts and themes based on the researcher’s interpretations. Overarching themes found via inductive coding were (1) operational benefits of routinely collected BFM data, (2) challenges in integrating and scaling BFM, (3) strategic role in reaching systematically missed populations, and (4) sustaining trust through BFM and closing the loop.

Initial coding was conducted by the first author. To support reflexivity and minimize bias, memos were kept during the process and code categories were regularly reviewed. Emerging themes were refined in consultation with the last author. For further quality assurance, a purposively selected subset of transcripts (n = 4), representing variation across stakeholder roles, was independently double coded by the first and second authors to assess consistency in code application. Coding discrepancies were discussed in analytic meetings and resolved by consensus, with refinement of code definitions where necessary. Thematic convergence across codes led to the development of key themes, which explored relationships between feedback mechanisms and the operational and strategic aspects of MDA delivery.

As this study drew on experiences from ASCEND-supported contexts, we were attentive to the potential influence of the research team’s professional proximity to the program. While familiarity with NTD implementation strengthened contextual understanding, analytic decisions were supported by structured coding procedures and collaborative theme review. Interviews were conducted by the second author with no prior involvement in ASCEND, and three of the five authors (second, third and fourth authors) were not directly engaged in ASCEND implementation, allowing for internal challenge and balanced interpretation.

## Results

### Perceptions on how to best operationalize BFM for MDA

Informants acknowledged the many methods of collecting feedback surrounding MDA that were introduced during the ASCEND program in 2020, and that have been used in subsequent programs since its closure. There was significant variation in how BFM were implemented. This variability reflected the adaptability of feedback mechanisms to local contexts and the different methodologies surrounding MDA delivery across settings. Informants recounted various methods of collecting feedback, including the methods mentioned in Fig 1.

In some regions, feedback was systematically embedded into existing tools, like coverage evaluation surveys. These included the Supervisors Coverage Tool (SCT), a small-scale survey conducted immediately following MDA where feedback could be used to improve upon the current MDA campaign, and the Independent Coverage Survey (ICS), a large survey that targets a larger population but is conducted within 3--6 months of MDA.

Other informal methods of collecting feedback, such as community meetings or daily post-MDA debriefs (CDD meetings), played a larger role. Informants acknowledged additional initiatives where feedback was collected through social media, anonymous dropboxes, and non-anonymous daily MDA logs. Informants explained that while dropboxes would yield many comments which were at times not even relevant to MDA, critical information occasionally emerged through these channels which helped programs adjust elements of the MDA.

Reflecting on the outcomes of the various mechanisms for collecting feedback, informants most often acknowledged CDD meetings, where the CDDs would review feedback collected during the campaign and share among each other and with the MDA supervisors and program staff, as a standout opportunity for collecting BFM:

“During MDAs, there were opportunities for CDDs to collect informal feedback while distributing drugs or when they convened at the end of the day to share their experiences and challenges… CDDs were pivotal in both delivering MDA and collecting feedback. In many cases, their interactions with communities during [drug] distribution allowed them to pick up on subtle issues, like hesitancy due to misinformation or logistical problems in reaching certain households. However, follow-up was often limited, which reduced the impact of this feedback.” – PI6.

CDDs were praised for their capacity to identify pivotal local issues as members of the communities where drugs were distributed, but limitations were noted regarding whether feedback was consistently documented during the meetings. Some informants believed that the lack of procedure in documenting the feedback was a missed opportunity which may have inhibited follow-up to the feedback received.

While discussing the best methods for ensuring operationally useful feedback, informants felt that BFM collected in a timely manner, or received during the MDA campaigns and immediately passed on to supervisors, had the best chance of being acted upon. Among these methods of collecting timely feedback, CDD meetings, along with the SCT survey, were cited as approaches that could have the quickest impact, allowing information to flow directly from community members up to supervisors, who were present and often facilitating the meetings. An informant explained how this was useful in their program:

“Supervisors or program leads would often review feedback gathered by CDDs in daily meetings, and if issues came up, like a household needing different hours of outreach or community concerns about side effects, we could immediately adjust plans for the next day’s activities.” – PI6.

The coverage evaluation surveys, both the SCT and ICS, were regarded as a good way to systematically integrate collection of more large-scale feedback in a way that was perceived by informants as less anecdotal than CDD meetings. However, as the ICS operates on a longer time scale, often taking six months to a year from the time of the MDA to generate and analyze results, they would only be helpful in improving subsequent campaigns. Some informants also felt that the data from these surveys was important for supplementing what was discussed in meetings:

“Independent surveys brought an external lens to the challenges communities faced: like fears about side effects or logistical barriers to access. These surveys sometimes uncovered issues not visible in the routine data collected by CDDs or supervisors.” – PI1.

Informants felt that another potentially impactful opportunity for discussing feedback was supervisory meetings, which were often conducted after the MDA campaign had concluded. Here, supervisors and other staff members involved during the MDA convene to discuss their experiences during the MDA campaign. Informants felt that the systematic integration of BFM in the discussion agenda would be very beneficial given the supervisors’ decision-making power. Informants reflected, however, that like with ICS surveys, these changes would only be able to go into effect in subsequent campaigns.

In discussing *how* changes could be best operationalized, informants discussed the importance of having a systematic process, from integrating the collection of feedback through analysis, monitoring, and ultimately reviewing key feedback to make tactical adjustments to the MDA where necessary.

“The national NTD program is in charge of guiding all levels if there is any change you have to conduct... For example, they may come up with some feedback during data review meetings. They look at the challenges and experiences from the previous MDA, and this feedback helps to adjust for the next MDA. Whether it’s changing data tools, adjusting the template, or modifying the approach for supervision, the program reviews feedback to ensure improvements are made each year.” – MS1.

“… it’s essential to have a way to ensure that feedback isn’t just collected but also acted upon. The country teams were usually good at managing this, but sometimes the resources weren’t sufficient to close the loop effectively.” – F2.

Key informants also revealed that various feedback collected often went by different names depending on the context. For example, in some settings, it was referred to as “community monitoring” or “health worker insights,” emphasizing how terminology and practices varied based on local needs, program design, and stakeholder involvement. A programmatic informant discussed alternative framings for feedback mechanisms in their program:

“We definitely used oral, written, and digital engagement mechanisms, but they were often framed more broadly as tools for outreach or engagement rather than strictly [as] feedback [mechanisms].” – PI2.

### Strategic role of BFM in improving drug coverage and reaching systematically missed populations

Informants were asked how feedback received could best be used to improve upon elements of MDA delivery. Most felt that such feedback could help programs tailor elements of their MDA strategies to better fit the local context, thereby facilitating the highest possible drug coverage among populations at risk.

“At the moment that the information comes through in the timeline of MDA or mop-up, you are able to tailor your community mobilization strategy based on what you have heard. For example, if coverage was low in one area, supervisors and CDDs could immediately adjust their approach to address the reasons behind low compliance.” – PI1.

“During the five days of MDA, there was daily feedback with supervisors who would review data from the previous day and tailor strategies to address issues like refusals or low coverage.” – PI1.

Some informants cited examples of how the integration of novel BFM had already been programmatically useful, in some cases helping to find additional people who were missed during the same round of MDA:

“During mop-up activities, we found households that were not reached during the initial MDA despite being reported as covered. This was revealed through feedback during supervision, which then allowed CDDs to go back and administer the drugs.” – MS2.

Furthermore, some informants felt that use of BFM could help programs understand why sub-optimal coverage in specific populations within communities was occurring, potentially providing insights into why some individuals and groups were routinely left out of MDA. These included understanding community dynamics like gender and language barriers:

“There were other barriers, like needing permission from males for women to engage, or people not understanding due to language barriers. These are the things you uncover when you do root-cause analysis. The BFM really helped document this and reveal what we might not have seen otherwise.” – MS2.

Multiple informants referenced that feedback received helped programs to directly address drivers of non-participation in MDA, such as fear of adverse events:

“In [one country], an area reported low compliance because of rumors that MDA is killing people. This originated from an unrelated incident where a woman died during a previous MDA. Feedback mechanisms allowed us to identify this misconception and address it directly with the community.” – PI1.

### Perceptions on challenges in integrating and scaling use of BFM

Several key informants flagged two critical issues in monitoring and evaluation of feedback that could inhibit operational changes. The first is that there is no platform where BFM data can be systematically reviewed and acted upon, as there is with other MDA data. For many countries that participated in ASCEND program, significant efforts have been made to streamline data entry and reporting to the District Health Information System 2 (DHIS2) platform. Informants explained that while there are many quantitative data fields on the MDA and its drug distribution performance, none exist for BFM:

“With DHIS2, the issue has always been that it captures quantitative data quite well, things like coverage, treatment numbers, but it doesn’t really have fields or a framework for qualitative feedback. This is where BFM insights are often lost, as there’s no standardized way to input those observations into DHIS2 or link them back to program adjustments. We’ve been advocating for updates to the system, but it’s a challenge.” – MS1.

The second issue is the lack of human resources for reviewing BFM data. Informants reported that there were no dedicated personnel investigating BFM results, which they believed limits the capacity for systematic monitoring and subsequent streamlining into the decision-making process.

### Sustaining community trust through BFM and closing the loop

Informants believed that community engagement was important for MDA campaigns and NTD control beyond simply its operational benefits. They also felt BFM could support community engagement and lead to more sustainable program delivery.

“[engagement of marginalized groups beyond the easy to reach target population] was really quite high on the agenda at the time, especially for countries where they were kind of close to elimination and eradication of diseases. Certainly, to maintain that momentum, there was a real […] need to maintain engagement. And really […] keep people on board to reach that last mile.” – PI6.

“Community sensitization and outreach are critical. It’s not just about distributing drugs; it’s ensuring that the community understands the importance of taking them and trusts the safety of the medication. If the community is informed and engaged, the coverage rates improve, and reinfection cycles are reduced.” – PI1.

Informants further detailed how they felt collecting feedback could support the essential task of keeping communities engaged with the MDA program:

“BFM are not just a way to get feedback; it’s also a way to foster engagement. When communities see their input leading to real changes, it motivates them to stay involved.” – MS2.

At the time of the interviews, some informants felt that community engagement was especially important due to community fatigue, which they felt was threatening continued participation in programs. Informants described how fatigue was both an issue for CDDs and community members.

“Some communities show resistance over time, feeling fatigued by the repeated MDAs, particularly if they don’t see tangible results or benefits. This requires more focused engagement and communication to explain the importance of continuing the interventions.” – PI4.

“Fatigue among CDDs after many years in the program is a real issue. They sometimes manipulate data or report good coverage without actually reaching all households, which we catch during data audits or supervision.” – MS2.

In the ASCEND BFM approach, “closing the loop”, or informing community members of the changes that had been made in the past from feedback, was the final stage of the BFM process. When asked about perceptions regarding closing the loop, informants agreed that it was a necessary part of the process.

“The value of the feedback mechanism is to build that trust and value people’s voices and give them a stake in what’s happening. In doing that, they may be more willing to engage, participate, and encourage others to take up services. So, if you don’t close the loop—if you gather information, act on it, and fail to tell people what you’ve done—you’ve broken the(ir) trust, and it doesn’t work.” – R2.

As with the methods of collection and operationalization of BFM, there were mixed opinions about how the loop should be effectively closed. Informants agreed that ‘closing the loop’ should be communicated through trusted and appropriate channels for the specific communities.

“Feedback should be delivered back to the community in ways that make sense to them—like through trusted messengers, community meetings, posters, or local radio.” – R2.

“We spent money on that campaign, on radio, on cars that are working in the neighborhoods... and they say we don’t hear radio a lot... Here the boxes or the cars in the neighborhood, we trust them. So, we know that next time... we invest in cars and this people that are going around.” – MS1.

The MEL specialist commented that one community did not listen to the radio but was rather accustomed to loudspeakers mounted on local cars, which resulted in wasted resources from programs on radio messages that failed to reach community members. This example of communication channels, used to close the loop, must be trusted, appropriate for the local context, and accessible to community members.

## Discussion

Results from this study indicate that professionals involved in different aspects of MDA delivery perceive BFM as a useful tool that may enhance MDA coverage, responsiveness, and program sustainability. Informants acknowledge potential benefits in systematically integrating BFM into various parts of the MDA process, while also acknowledging limitations that may challenge its use in various NTD programmatic settings. Table 2 synthesizes the core themes identified in the Results section and presents corresponding operational recommendations derived directly from these findings. Several of the operational examples discussed below draw from ASCEND supported efforts to formalize beneficiary feedback within routine MDA delivery. As ASCEND activities were implemented within WHO-recommended preventive chemotherapy frameworks, they provide an illustrative case through which broader lessons on integrating BFM into MDA systems can be examined. As this study draws on stakeholder perceptions rather than measured program outcomes, findings should be interpreted as indicative of potential pathways rather than demonstrated causal effects.

**Table 2:** Summary of perspectives on optimal BFM mechanisms and key limitations.

Theme	Key Finding	Recommendation
**Perceptions on BFM collection methods**	There are many methods of collecting BFM that countries have used, but there was consensus around the utility of taking advantage of existing programmatic structures, especially daily CDD-supervisor meetings, which were seen as effective venue for BFM to yield timely and actionable data.	Systematically integrate BFM into all daily CDD meetings (and supervisory meetings) as part of MDA microplanning and implementation protocols.
	Coverage evaluation surveys offer further opportunities of systematic collection of feedback directly from a large sample of community members.	Ensure coverage surveys include feedback elements and are conducted regularly with sufficient funding, standardize questions for BFM that yield actionable data.
**Structural challenges in existing systems**	Platforms for digitally entering and monitoring MDA data, like DHIS2, do not currently accommodate qualitative or BFM-specific fields, limiting feedback capture.	Advocate for integration of qualitative and BFM-specific fields into DHIS2 or complementary digital platforms.
	There is often no designated role or unit within the MoH responsible for ongoing BFM monitoring or coordination.	Create designated BFM focal points within national NTD programs to manage and act on community feedback.
**BFM to Improve Coverage & Reach Missed Populations**	Feedback helps identify areas and groups consistently missed in MDA, such as due to refusals, absenteeism, or CDD coverage gaps.	Use BFM data to map and re-target persistently missed or refusing populations in real-time during MDA rounds.
	Informants widely agreed that BFM can directly contribute to improving coverage by enabling course corrections.	Train MDA teams to analyze BFM data between rounds and adjust sensitization or distribution strategies accordingly.
**BFM and Program Sustainability**	Informants believed that closing the loop by reporting findings back to the community and involving them in planning enhances trust and engagement.	Develop standard processes to share feedback findings with communities post-MDA to enhance transparency and trust.
	Feedback systems supported community leadership and ownership, seen as key to long-term sustainability of NTD efforts.	Involve community leaders in review and planning processes to build ownership and long-term support for MDA programs.

Informants agreed that BFM can be integrated into MDA program delivery through a variety of methods, of which CDD meetings and SCT surveys were highlighted as practical and timely sources of actionable feedback, and the ICS, despite its slower data turnaround, as a good channel to routinely collect large amounts of feedback. As the integration of BFM in MDA is relatively novel, identifying and documenting the best methods of delivery is an important step in creating an evidence base for other programs to draw from. The results of this study were concordant with a 2022 study on the integration of BFM into the ASCEND West countries, which also highlighted the value of integrating BFM into coverage surveys and CDD meetings [18]. That study added the value of supervisory reports, a written record from CDD meetings, where key issues to arise could be escalated to supervisors. Currently, much of the information surrounding the best methods of integrating BFM into MDA activities exist in gray literature, which includes reports for national programs, funding organizations, NGOs, and implementing partners.

Informants emphasized that integrating BFM into existing tools, rather than creating entirely new platforms, would improve the feasibility of integration into resource-constrained programs. This aligns with broader evidence from NTD control programs suggesting that leveraging existing supervisory meetings and coverage surveys, rather than integrating new tools, can reduce operational burden, avoid duplication, and make better use of personnel and resources [41]. Continued use of BFM in routine programming enables programs to refine and streamline both the methods of collection and questions used in existing tools based on their demonstrated utility and actionability.

Currently, the WHO guidelines do not offer extensive recommendations on integrating feedback mechanisms. Contextualized coverage evaluation surveys are mentioned in the WHO field manual on “Preventive Chemotherapy: Tools for improving the quality of reported data and information,” and include some aspects of BFM, like knowledge, attitudes and practices (KAP) questions, or questions on reasons for non-compliance [3]. However, these are limited and not prioritized as a key component of the tool. As research has demonstrated, country programs may wait for standardized WHO guidance and recommendations in their treatment guidelines before integrating novel tools into their workflow [42]. Global leadership may play a role in standardizing and strengthening BFM practices by integrating them into preventive chemotherapy as well as monitoring and evaluation guidelines. Since some informants posited that a one-size-fits-all approach may not be suitable for BFM, guidance documentation will have to balance advocating for BFM based on best practices while maintaining flexibility in the methodology used to meet the specific needs of communities.

Key informants and WHO preventive chemotherapy guidelines recognize achieving high, uniform, and equitable drug coverage during MDA as one of the core operational goals and a key indicator for programmatic success [3], particularly as some settings transition into ‘last mile’ or ‘elimination stage’ planning [43]. Informants perceived BFM as a tool that could help programs improve drug coverage, both in the short term through actionable operational insights to reach missed groups, and in the long term by promoting program sustainability through community involvement that encourages future participation. Informants cited examples where BFM revealed gendered barriers, linguistic mismatches, religious concerns, or persistent misinformation that had previously gone undetected in review of routine program data.

Within the theme improving coverage and reaching missed populations (Table 2), informants described BFM as a potentially useful tool for better understanding subsets of the population that were not currently participating in MDA. Among these populations are “never-treated” individuals, a group considered increasingly programmatically important to address to reach targets in reduction of disease progression and transmission [44–46]. Past research has demonstrated that community engagement and feedback can be effective for understanding the barriers among populations left out of previous MDA campaigns, and ultimately improve drug coverage [10,47]. These perceptions are concurrent with a growing body of evidence demonstrating that when part of MDA programs, collection of community feedback and integration of participatory approaches can help improve drug coverage in communities [14,48,49]. These perceptions suggest potential programmatic value and may warrant consideration of BFM integration.

Given the multi-year cyclical nature of MDA, informants agreed that building trust among communities was essential for ensuring program sustainability over time. Consistent with the theme on sustaining trust and closing the loop in Table 2, BFM were perceived by informants as a promising entry point for building that trust, especially in programs where community involvement had been historically limited. This is especially important given CDD and community member fatigue from repeated rounds of MDA, which was discussed by informants and echoed in literature as an emerging threat to reaching disease control targets [25]. For diseases like schistosomiasis, where MDA may not effectively lead to local elimination of disease transmission, and thus require many rounds of successive MDA campaigns for morbidity control [50], maintaining community trust to keep participation high is integral. While studies have demonstrated that the integration of community feedback and engagement strategies can contribute to long term MDA program acceptance [51], further research is required across preventive chemotherapy NTD programs to determine how these strategies can be implemented in practice. Informants also highlighted the importance of “closing the loop”, or ensuring communities are informed of how their feedback has shaped programming. Despite some evidence on the importance of closing the loop [18,24], there remains limited evidence on the best methods for doing so.

Understanding the barriers that may hinder the successful integration of BFM into routine MDA is integral. As identified in the Results under structural challenges, informants highlighted challenges related to monitoring of the feedback that was collected, namely: 1) that there was not an existing platform to monitor feedback, and 2) NTD programs lacked dedicated staff to monitor the feedback and ensure that it was acted upon. For informants who had worked in ASCEND East countries, this was specifically in reference to the DHIS2 platform on NTDdata.org that had been established under the program to monitor key indicators. While advances such as the implementation of digital data entry systems for MDA activities, such as DHIS2, has been an integral improvement to allow programs to improve access to key MDA monitoring data [52], these platforms typically lack fields for qualitative feedback as well as indicators related to BFM. Some BFM indicators can also be made quantitative to facilitate analysis of large surveys, as with questions asked in the SCT and ICS surveys [19,20,53]. As for the informant perspective on lack of dedicated staff, this could just as well be referring to a lack of M&E staff that are trained to review BFM rather than a deficit in human resources. Staff who are monitoring drug coverage can be trained to look at BFM data and examine associations between community perspectives surrounding the MDA and drug coverage.

### Strengths and limitations

A key strength of this study lies in the potential for transferability of its findings across NTD programs implementing MDA. Additionally, the inclusion of perspectives from stakeholders across diverse professional roles and geographic settings further strengthens the relevance of these findings to similar MDA delivery environments.

This study has two limitations. First, recruiting participants from within our existing NTD network may have introduced the possibility of selection bias. Prior exposure to BFM was a prerequisite for participation, which may have influenced the responses and skewed discussion toward conceptual alignment or optimism regarding BFM potential. However, the interviewers sought to mitigate this by attempting to maintain objectivity throughout the research process, including during question formulation, interviewing, and analysis. Second, these findings reflect the perceptions of key informants rather than direct observations or community perspectives, which may constrain insights into how BFM are perceived at the community level.

### Recommendations

As MDA is an opportunity not just to distribute drugs but engage with the community, informants recommended that BFM be integrated into existing MDA structures and used consistently to gather timely and actionable insights. For maximum impact, feedback should be analyzed by dedicated staff and included in digital monitoring platforms. The authors recommend that this should be a task for M&E staff who are also looking at drug coverage, to make appropriate associations between community perspectives and drug coverage. Ensuring publication in peer reviewed journals, not only in project reports and other forms of gray literature, can improve visibility and bolster the evidence base to support the use of BFM by national programs. Crucially, community members should be informed about how their feedback is used to ensure transparency, foster program ownership, and strengthen participation.

While these recommendations reflect informant perspectives on strengthening BFM integration, their implementation must be considered within the realities of resource constraints, workforce capacity, and competing program priorities in many endemic settings. Phased integration leveraging existing tools and M&E personnel may improve feasibility, particularly in lower-capacity contexts.

We recommend conducting formal experimental trials that seek to provide empirical evidence of the capacity for BFM to improve drug coverage, which will be fundamental to convince NTD program leadership in both international and national health programs of its value. Such trials should include comparison arms between programs implementing BFM and those that do not, using clearly defined approaches based on successful methods identified in this and similar studies, and be conducted over multiple years to assess sustained adherence. Future research should incorporate direct community perspectives alongside professional stakeholder insights, for example through focus group discussions, participatory workshops, or mixed-method designs that combine qualitative inquiry with coverage data. Integrating community voices will likely be integral to validate, refine, or challenge professional interpretations of BFM effectiveness and to ensure that feedback mechanisms remain responsive to those they are intended to serve.

We furthermore recommend that the WHO work to standardize terminology for BFM, methods of data collection and key indicators, and integrate these components into preventive chemotherapy distribution guidance, guidelines for coverage evaluation surveys and broader M&E frameworks. Future empirical studies should inform the development of standardized BFM questions, reporting metrics and practical guidance on effectively closing the loop with communities.

## Conclusions

Drawing from stakeholder perspectives, several foundational elements emerge as essential for integrating BFM within routine MDA delivery: embedding feedback collection within existing program structures, assigning clear responsibility for data synthesis and review, establishing mechanisms to translate feedback into programmatic adjustments, and institutionalizing processes for communicating responses back to communities.

Given their perceived potential for improving drug coverage, program sustainability through community involvement, and equitable inclusion of systematically missed populations, BFM merits serious consideration in MDA campaigns across preventive chemotherapy NTDs. Integration of tools like BFM warrants formal evaluation to determine whether they improve cost effectiveness and accelerate progress toward elimination targets. BFM may represent a valuable addition to the arsenal of tools that seek to optimize delivery of MDA and hopefully facilitate the reduction of the considerable burden of disease caused by the five PC-NTDs.

## Supporting information

S1 File Interview Guide. (DOCX)
