# Supplementary material for: Beneficiary feedback mechanisms: Exploring ways to systematically integrate community feedback in mass drug administration delivery for NTDs
Source: PLoS Negl Trop Dis. 2026 Jun 8;20(6):e0014373. doi: 10.1371/journal.pntd.0014373 (PMC13258146; doi:10.1371/journal.pntd.0014373)
Supplement: S1 File — (DOCX) [file pntd.0014373.s001.docx]

# Annex: Interview guide

*This interview guide gives an indication of questions and topics. Specific questions will be adjusted for different participants.*

- **Introduction**
- Through this interview we hope to gain a better understanding of the use of community feedback mechanisms in MDA programmes, and how they can best be operationalized to improve MDA programmes. Therefore, we will first discuss your role in MDA programmes and your professional knowledge of community feedback mechanisms. Then we dive deeper into the specific operational aspects of community feedback, whereafter we discuss contributing factors. In the end we will discuss your professional perception towards the usefulness and towards the future of community feedback.
- Before we start, I would like to ask your consent on the recording of this interview and to confirm that you understand and agree with the informed consent form. The recordings and transcriptions will be anonymized and stored safely. In addition, you should know that you can withdraw from this interview at any moment and that you are free to skip questions you consider discomfortable or irrelevant. Do you have questions before we start?
- **Current programme organization**
- *First some general questions about involvement, purpose and motivation.*
- Can you describe your professional occupation and for how long you have been in this position?
- How are you currently involved in MDA programmes?
- Can you describe your role and responsibilities?
- What do you know about any community feedback that was ever collected in MDA programming?
- Why do you think community feedback is important to incorporate in MDA programming?
- Do you have an idea of what community feedback mechanisms in MDA programmes could look like?
  - Can you explain? Introduce how we understand community feedback.
- **Operational capacity**
- *Part II dives deeper into the specific operational aspects of community feedback: how does community feedback work in the participants’ context, who are involved and via what tools is feedback collected.*
- **Service and workforce**
- Who are/were involved in the implementation and execution of community feedback on national, district and community level?
- How are the streams of communication and flow of information constructed?
- Who appointed the roles to these specific persons and on what basis?
- *Introduce visualization on BFM loop*
- *In theory, community feedback consists of 6 steps that form a constant loop between the community and the one’s being able to make a change in the MDA programme, as visualized below. We are interested to see if and how these steps are organized in your activities.*
  - - - Feedback given
      - Feedback acknowledges
      - Analysis/referral
      - Response/action (or no action)
      - Communicate response
      - Awareness and confidence to give feedback in the future
- Figure: Feedback loop
- *[ step feedback given ]* How is feedback collected from the community?
- Who is responsible, via what tools, in which capacity (paper/electronic etc)?
- What specific information/feedback is asked from the community members?
- Are the persons involved in the execution of community feedback trained/prepared in collecting feedback / skilled to collect feedback?
- *[ step feedback acknowledged ]* What happens with the collected feedback?
- Where is that information/ community feedback stored after collection?
- How is it communicated to the person responsible for monitoring and evaluation of feedback?
- How are communication streams formulated?
- What kind of information would you ideally like to receive from the community with the implementation of community feedback mechanisms?
- What information do you think would be useful for improving programmes? Do these questions exist? Why not?
- How would you like to see the community feedback be routinely implemented
- How would you like to see community feedback integrated into existing health systems/MDA programmes?
- By whom would you like to see the programme operated?
- **Monitoring & evaluation**
- *[analysis / referral]* How is the collected feedback monitored and evaluated?
- On what basis, who is responsible?
- Based on what information is feedback appointed as valuable? And who determines its value?
- What happens with feedback appointed as valuable?
- How is valuable feedback reported?
- *[ Response ]* How is evaluated feedback acted upon?
- Who is responsible for acting upon evaluated feedback?
- Perceive implementers this information as valuable?
  - Are the changes made being communicated back to the community? How?
  - What do you think can be done to make the evaluated feedback of more value to the implementers?
    - Are the feedback questions asked to the community not valuable? How should the feedback questions be changed?
    - Does the monitoring & evaluation team not have the right understanding of ‘valuable’?
    - Are the recommendations made based on evaluation not ‘valuable’?
- *[Communicate response]* Is performance of community feedback monitored and if yes, how?
- On the basis of what outcome indicators, which information and who is responsible for this?
- **Operational changes from feedback**
- As a result of community feedback, what changes have been made in MDA programming?
- Or what stayed the same because of positive feedback? Can you give examples?
- In the process of collecting community feedback, are there tools that are better at collecting valuable feedback than others?
- Why? What factors play a role in this?
- In previous rounds of community feedback, which aspects worked well and which did not work well?
- Why? Can you give examples?
- **Contributing factors**
- *After specific operational aspect of community feedback, we zoom out to other aspects of the process.*
- **Policy and planning**
- Who or what motivates MDA programmes to include community feedback mechanisms?
- How and why?
- Are you aware of specific (national NTD health) policies or guidelines describing the implementation of community feedback in MDA programmes?
  - Who is responsible for the implementation of these guidelines?
  - Would specific policies or guidelines help for implementation?
- How would you describe the role that donors play in the implementation of community feedback mechanisms in MDA programmes?
- **Sustainable implementation / information systems**
- How do you think community feedback can be sustainably implemented in MDA programs?
- Is the collection of community feedback integrated in the existing health system, how and/or does it align with the governmental programme?
- Are the current health information systems (= *system that ensures production, analysis, dissemination and use of timely and reliable information about diseases)* for MDA suitable to also incorporate community feedback mechanisms?
- Are the outcomes of community feedback communicated to MDA programmes where community feedback is not (yet) involved?
- Are the responses to community feedback integrated in all MDA programmes (not only the one that provided the community feedback)?
- **(Financial) Resources**
- How is the use of community feedback in MDA programmes currently financed?
- Donors, domestic funding, national health budget?
- Are there additional costs for implementing community feedback when it is integrated in existing health systems?
- What additional resources did you need for implementing community feedback mechanisms?
- What (additional) resources do you think are essential in incorporating community feedback in MDA programming?
- **Perceptions**
- *Besides operational capacity and contributing factors, we are also interested to see how community feedback is perceived as useful and what barriers implementation can face.*
- **Usefulness and barriers**
- What do you think are the benefits of incorporating community feedback in MDA programmes?
- How do you think the community benefits from providing community feedback?
  - (Should enhance empowerment and accountability.)
- Can you think of any risks involved in the collection, analysis and response to community feedback?
- Which risks? Who is responsible for mitigating these risks?
- Can you think of barriers to overcome when incorporating community feedback in MDA programmes?
- Physical barriers like infrastructure (roads/public transport), financial barriers like funding, social barriers like fear, trust, personal preferences.
- How are these barriers tackled right now? / How can these barriers be overcome?
- Can you explain how minority groups are safeguarded when providing feedback?
  - ( Safeguarding minority groups (women want women e.g., or translators needed) )
- Do you think community feedback is valuable to improving MDA programmes?
- Do you think that it could help improve drug coverage e.g.? How?
- **Future**
- Currently, community feedback in MDA programmes has mainly had the purpose of improving delivery of drugs. Do you think community feedback can also be useful for other aspects of MDA programmes?
- **Conclusion**
- *To conclude this interview, what are for you the three most important lessons about community feedback in MDA programmes we have to take with us?*
- *Thank you for your time and valuable insights. Do you still have questions for us?*
