# Supplementary material for: Beneficiary feedback mechanisms: Exploring ways to systematically integrate community feedback in mass drug administration delivery for NTDs
Source: PLoS Negl Trop Dis. 2026 Jun 8;20(6):e0014373. doi: 10.1371/journal.pntd.0014373 (PMC13258146; doi:10.1371/journal.pntd.0014373)
Supplement: Attachment — Submitted filename: Reviewers FeedBack.docx [file pntd.0014373.s002.docx]

Comments for Authors

Thank you to the authors for presenting this timely and policy-relevant manuscript, which addresses an important gap in the operationalization of community engagement within mass drug administration programs for neglected tropical diseases. I can see that the focus on Beneficiary Feedback Mechanisms (BFM) is well aligned with current global priorities around equity, “Leave No One Behind,” and sustainable NTD elimination. The manuscript is generally well written, conceptually coherent, and grounded in relevant programmatic experience.

The following could improve the clarity and positioning of the research contribution

- First at the Abstract and Background, (lines ~22–24; ~153–159), while the manuscript clearly identifies a gap in evidence regarding Beneficiary Feedback Mechanisms (BFM) in MDA programs, the novel contribution of this study could be stated more explicitly. The authors should strengthen the manuscript by clarifying how this study advances existing knowledge beyond prior ASCEND reports and related qualitative studies, particularly in terms of conceptual, operational, or implementation insights.

- For the justification of geographic and programmatic scope at methods – Participant selection, (lines 172–179), the focus on ASCEND-supported countries in sub-Saharan Africa is appropriate; however, the manuscript would benefit from a clearer justification of how findings may (or may not) be transferable to non-ASCEND contexts or other NTD programs. Explicit discussion of contextual boundaries would strengthen interpretability and external relevance.

- Looking at the sample size and saturation rationale (Methods – Sample size, lines 186–194), the decision to stop recruitment at 14 interviews is reasonable for qualitative inquiry, but the manuscript would benefit from a more explicit description of how thematic saturation was assessed, beyond recurrence of themes. Also, briefly clarifying whether saturation was assessed formally or iteratively would improve methodological transparency.

- Again, looking at the importance of reflexivity and positionality of the research team (Methods – Data analysis, lines ~218–222), while steps to minimize bias are mentioned (e.g., memos, double coding), the manuscript would be strengthened by a brief reflexivity statement describing the researchers’ positionality, professional proximity to ASCEND, and how this may have influenced interpretation. This is particularly relevant given the close linkage between the study context and the authors’ professional roles.

- In terms of greater methodological detail on coding framework at (Methods – Data analysis, lines ~211–225), readers would benefit from greater detail on the deductive framework used (e.g., whether it was informed by existing BFM frameworks, WHO guidance, or ASCEND tools). Clarifying this would enhance reproducibility and analytical rigor.

- For the distinction between perceptions and demonstrated effects at the Results; Discussion, lines ~423–476), the results compellingly describe informants’ perceptions of BFM utility; however, in several sections, the language could more clearly distinguish perceived benefits from demonstrated or measured outcomes. Strengthening this distinction would reduce the risk of over-interpretation and maintain analytical precision.

- Based on the balance between ASCEND-specific and generalizable findings particularly at the results and discussion, Given the centrality of ASCEND examples, the authors should consider explicitly separating ASCEND-specific operational insights from those likely applicable across NTD programs. This would help readers understand which findings are context-bound versus broadly informative.

- Integration of community-level perspectives at the Limitations section (lines 516–518)
The limitation regarding absence of direct community voices is appropriately acknowledged. The manuscript could be strengthened by briefly discussing how future research might integrate community perspectives alongside key informant insights, particularly to validate or challenge professional interpretations of BFM effectiveness.

- Presentation and synthesis of Table 2 (Table 2; Discussion, lines ~419–428)
Table 2 effectively summarizes findings and recommendations; however, the authors should consider more explicitly linking each recommendation back to specific empirical findings in the Results section, either through cross-referencing or clearer narrative signposting.

- looking at the recommendations and feasibility considerations (lines ~519–536)
The recommendations are well-aligned with findings, but feasibility considerations such as resource constraints, workforce capacity, and competing program priorities could be briefly discussed to contextualize implementation realism, especially in lower-capacity settings.

- Language precision and consistency throughout manuscript is noted and it is important that minor improvements should be made by ensuring consistent use of terminology (e.g., BFM vs. community feedback vs. participatory feedback) and avoiding occasional normative phrasing that may imply endorsement rather than analysis. This would further enhance analytical neutrality.

-The authors could strengthen policy relevance by more clearly articulating how their findings could inform specific elements of guideline development (e.g., indicators, minimum standards, accountability loops).

Overall Assessment

This manuscript addresses a critical and underexplored dimension of NTD program delivery and offers valuable qualitative insights from diverse stakeholders. With modest refinements to clarity, methodological transparency, and interpretive precision, the paper would make a strong contribution to the evidence base on community engagement and implementation science in NTD control.
