# Supplementary material for: Beneficiary feedback mechanisms: Exploring ways to systematically integrate community feedback in mass drug administration delivery for NTDs
Source: PLoS Negl Trop Dis. 2026 Jun 8;20(6):e0014373. doi: 10.1371/journal.pntd.0014373 (PMC13258146; doi:10.1371/journal.pntd.0014373)
Supplement: Attachment — Submitted filename: BFM Response to Reviewers_24Feb.docx [file pntd.0014373.s005.docx]

Dear editors and reviewers,

We would like to sincerely thank the Editor and Reviewers for their thoughtful and constructive feedback on our manuscript, *“Beneficiary Feedback Mechanisms: Exploring ways to systematically integrate community feedback in mass drug administration delivery for NTDs.”*

We are grateful for your review of our work and appreciate the detailed suggestions, which have helped us strengthen the clarity, methodological transparency, and interpretive precision of the manuscript.

Below, we respond to each comment point by point. All changes have been incorporated into the revised manuscript and are reflected in the version with track changes.

Sincerely, on behalf of all co-authors,

Jake Mathewson

| **General** **comments**  This is a timely and relevant implementation-focused qualitative study addressing an important operational gap in NTD programs: the systematic collection and integration of community feedback to improve MDA delivery. The manuscript is clearly written, based on a well-designed interview approach, and presents plausible and programmatically relevant findings. Its main strengths lie in its focus on an underexplored aspect of MDA implementation and its synthesis of stakeholder perspectives across multiple roles, contributing useful operational insights for NTD programs. | Thank you for this comment. |
| --- | --- |
| However, the manuscript’s novelty is primarily conceptual rather than empirical, and several methodological and interpretive issues require attention. **Greater transparency is needed regarding qualitative analytic procedures, including what constituted the analytic sample for double coding and how coding disagreements were resolved.** | Thank you for this important point.  We agree that additional detail regarding analytic procedures strengthens transparency. We have revised the Data Analysis section to clarify that a purposively selected subset of transcripts (n = 4), representing variation across stakeholder roles, was independently double coded.  We further specify that discrepancies were discussed in analytic meetings and resolved by consensus, with refinement of code definitions where necessary. |
| Claims of “external validity” appear overstated given the small, network-based sample largely drawn from ASCEND-supported contexts**; the findings are better framed as transferable rather than externally validated**. | Thank you for this important clarification.  We agree that “external validity” may overstate the nature of generalizability in a qualitative, network-based study. We have revised the manuscript to frame the findings in terms of transferability rather than external validation and have refined the Strengths and Limitations section to more precisely describe the contexts to which the findings may be applicable. We believe this adjustment strengthens the methodological precision of the manuscript. |
| In addition, some statements imply effectiveness (e.g., improved coverage or equity) based on anecdotal examples and **should be tempered to clearly distinguish perceived pathways from demonstrated outcomes**. | **Response to Reviewers #1 and #2 (regarding distinction between perceptions and demonstrated effects):**  We thank the reviewers for highlighting the importance of clearly distinguishing stakeholder perceptions from demonstrated or measured program outcomes.  As this study was designed as an exploratory qualitative inquiry, our findings reflect informant perspectives on the perceived utility and operational pathways of BFM rather than empirically measured effects on drug coverage, equity, or sustainability.  In response, we have made several revisions throughout the manuscript to strengthen this distinction. Firstly, we added a clarifying statement at the end of the first paragraph of the Discussion explicitly noting that findings should be interpreted as describing perceived mechanisms and potential pathways rather than demonstrated causal effects.  Second, we have tempered language in the Abstract, Results, Discussion, and Conclusions to ensure that statements implying effectiveness are clearly framed as stakeholder perceptions or areas requiring further empirical evaluation. Finally, we strengthened the Limitations section to more explicitly acknowledge that findings reflect stakeholder perspectives rather than direct community experiences or measured program outcomes.  We have carefully reviewed and revised the manuscript to ensure this distinction is applied consistently throughout and are happy to make any further refinements if needed. |
| Finally, **clearer specification of minimal or essential components of beneficiary feedback mechanisms within MDA** would strengthen the paper’s practical contribution. | Thank you for this constructive suggestion.  While the operational elements of BFM integration are discussed throughout the Results and Discussion, we have revised the Conclusion to more explicitly synthesize the foundational components identified through stakeholder perspectives.  Specifically, we have now added a brief section in the Conclusions outlining key elements of feedback collection within existing program structures, assigning clear responsibility for data synthesis and review, establishing mechanisms to translate feedback into programmatic adjustments, and institutionalizing processes for communicating responses back to communities (lines 702-706). |
| **Background** |  |
| N/A |  |
| **Methods** |  |
| The objectives are clearly stated and appropriate for an implementation-focused qualitative study. A qualitative, key-informant interview design is appropriate for exploring stakeholder perspectives on beneficiary feedback mechanisms in MDA delivery. The study population (implementers, donors, and technical partners) is clearly described and relevant to the objectives. | Thank you for this comment. |
| However**, findings reflect stakeholder perceptions rather than direct community or beneficiary experiences**, which **should be more clearly** emphasized. | Please see comment above: **Response to Reviewers #1 and #2 (regarding distinction between perceptions and demonstrated effects):** |
| For qualitative research, the sample size (14 informants) is acceptable, but the manuscript would benefit from clearer justification regarding saturation and representation across stakeholder groups. | Thank you for this constructive comment.  We have revised the Methods section (Participant and Sample Size subsection) to clarify the justification for the 14 interviews. The text now explains that sample size was guided by thematic saturation and information power, with supporting references. We indicate that thematic recurrence was observed during data collection and confirmed during coding and analysis. We also clarify how the sample captured key institutional perspectives, noting that participants in smaller stakeholder categories held cross-country or oversight roles. |
| **Results** |  |
| The analysis presented is generally consistent with the stated qualitative analytic approach, but additional detail on the execution of the analysis plan (e.g. sampling for double coding and resolution of coding discrepancies) would be helpful, to improve transparency. | Thank you for reiterating this important point.  As noted in our response above, we have revised the Methods (Data Analysis) section to clarify this issue and have addressed it in detail in the corresponding response. We believe these revisions fully resolve the concern. |
| The results are clearly written and logically organized, with key themes presented in a coherent manner, although… | Thank you for this feedback. |
| some conclusions would benefit from clearer separation  between perceived mechanisms and demonstrated outcomes. | Please see comment above: **Response to Reviewers #1 and #2 (regarding distinction between perceptions and demonstrated effects):** |
| The tables and figures are of generally good quality and support the text. | Thank you for this feedback. |
| Conclusions |  |
| Overall, the conclusions are generally supported by the qualitative data presented, particularly with respect to stakeholder perceptions of the value and feasibility of beneficiary feedback mechanisms in MDA delivery.  However, **some conclusions—especially those implying improvements in coverage or equity—would benefit from clearer qualification to distinguish perceived pathways from empirically demonstrated effects.** | Please see comment above: **Response to Reviewers #1 and #2 (regarding distinction between perceptions and demonstrated effects):** |
| The authors do acknowledge several key limitations, including potential selection bias and the lack of direct beneficiary perspectives, **though these limitations could be more prominently emphasized**. | Thank you for this comment.  We agree that clearly emphasizing these limitations strengthens transparency. In response to related reviewer feedback, we have expanded the Strengths and Limitations section to more explicitly discuss potential selection bias resulting from recruitment within our existing NTD network and the requirement for prior exposure to BFM. We have also strengthened discussion of the absence of direct community perspectives and added a forward-looking recommendation outlining how future research could integrate community voices alongside professional stakeholder insights. We believe these revisions more prominently and clearly emphasize the study’s limitations while clarifying the scope of the findings. |
| The manuscript clearly discusses how the findings can inform implementation practice and program design, and the public health relevance is well articulated, given the importance of community engagement for effective and equitable NTD control and elimination programs. | Thank you for this feedback. |

**Reviewer #1**

**Reviewer #2**

| **General Comments** |  |
| --- | --- |
| This manuscript addresses a critical and underexplored dimension of NTD program delivery and offers valuable qualitative insights from diverse stakeholders. With modest refinements to clarity, methodological transparency, and interpretive precision, the paper would make a strong contribution to the evidence base on community engagement and implementation science in NTD control. | Thank you for this feedback. |
| Thank you to the authors for presenting this timely and policy-relevant manuscript, which addresses an important gap in the operationalization of community engagement within mass drug administration programs for neglected tropical diseases. I can see that the focus on Beneficiary Feedback Mechanisms (BFM) is well aligned with current global priorities around equity, “Leave No One Behind,” and sustainable NTD elimination. The manuscript is generally well written, conceptually coherent, and grounded in relevant programmatic experience. | Thank you for this feedback. |
| **Abstract and background** |  |
| First at the Abstract and Background, (lines ~22–24; ~153–159), while the manuscript clearly identifies a gap in evidence regarding Beneficiary Feedback Mechanisms (BFM) in MDA programs, the novel contribution of this study could be stated more explicitly. The authors should strengthen the manuscript by clarifying how this study advances existing knowledge beyond prior ASCEND reports and related qualitative studies, particularly in terms of conceptual, operational, or implementation insights. | Thank you for this valuable suggestion.  We agree that more explicitly articulating the manuscript’s contribution strengthens its positioning within the existing literature. In response, we have revised both the Abstract and Background to clarify how this study advances knowledge beyond prior ASCEND reports and related qualitative work.  Specifically, we now emphasize that this study provides operational insight into how BFM are integrated within routine MDA programming, drawing on cross-stakeholder interviews to analyze their perceived value, practical applications, and implementation challenges. We also clarify how these findings contribute transferable implementation insights relevant to preventive chemotherapy programs operating beyond the ASCEND context. |
| For the justification of geographic and programmatic scope at methods – Participant selection, (lines 172–179), the focus on ASCEND-supported countries in sub-Saharan Africa is appropriate; however, the manuscript would benefit from a clearer justification of how findings may (or may not) be transferable to non-ASCEND contexts or other NTD programs. Explicit discussion of contextual boundaries would strengthen interpretability and external relevance. | Thank you for this important suggestion.  We agree that clarifying the scope and focus strengthens interpretability. We have revised the Methods (Participant Selection) section to explicitly describe the ASCEND-supported implementation context and to clarify that the findings are most directly applicable to preventive chemotherapy NTD programs operating under similar MDA delivery structures.  We also note that transferability to non-preventive chemotherapy NTD programs or substantially different health system contexts should be interpreted cautiously. |
| **Methods** |  |
| Looking at the sample size and saturation rationale (Methods – Sample size, lines 186–194), the decision to stop recruitment at 14 interviews is reasonable for qualitative inquiry, but the manuscript would benefit from a more explicit description of how thematic saturation was assessed, beyond recurrence of themes. Also, briefly clarifying whether saturation was assessed formally or iteratively would improve methodological transparency. | Thank you for this helpful suggestion regarding methodological transparency.  We have revised the Methods section (Participant and Sample Size subsection) to provide a more explicit account of how thematic saturation was assessed. The manuscript has been revised to clarify the rationale for discontinuing recruitment. We now state that recruitment was discontinued after the fourteenth interview, as successive interviews reinforced previously identified themes and did not generate substantively new codes. This decision was consistent with principles of thematic saturation and information power guiding the study’s sample size, cited in the additions. |
| Again, looking at the importance of reflexivity and positionality of the research team (Methods – Data analysis, lines ~218–222), while steps to minimize bias are mentioned (e.g., memos, double coding), the manuscript would be strengthened by a brief reflexivity statement describing the researchers’ positionality, professional proximity to ASCEND, and how this may have influenced interpretation. This is particularly relevant given the close linkage between the study context and the authors’ professional roles. | Thank you for this suggestion. We agree that clarifying positionality strengthens transparency in qualitative research.  We have added a brief reflexivity statement to the Data Analysis section acknowledging the study’s ASCEND-supported context and the research team’s varying degrees of professional proximity to the program. We clarify that 1) while familiarity with NTD implementation strengthened contextual understanding, interviews were conducted by a researcher with no prior involvement in ASCEND, and 2) that three of the five authors were not directly engaged in ASCEND implementation.  We also note that collaborative theme review and structured coding procedures were used to support balanced interpretation. We believe this addition enhances methodological transparency and addresses the potential influence of contextual proximity. |
| In terms of greater methodological detail on coding framework at (Methods – Data analysis, lines ~211–225), readers would benefit from greater detail on the deductive framework used (e.g., whether it was informed by existing BFM frameworks, WHO guidance, or ASCEND tools). Clarifying this would enhance reproducibility and analytical rigor. | Thank you for this helpful comment.  We agree that providing greater clarity regarding the deductive component of the hybrid analytic approach strengthens the methodological transparency of the manuscript. We have revised the Data Analysis section to explicitly describe the sources that informed the deductive coding framework.  Specifically, we now state: The deductive component was informed by prior operational literature on beneficiary feedback mechanisms, including FCDO-supported program reports, as well as established implementation frameworks such as the USAID NTD Sustainability Framework, which outlines key dimensions of NTD program delivery and sustainability. These sources provided sensitizing concepts that structured early coding while allowing themes to evolve inductively through iterative transcript review.” |
| **Results** |  |
| For the distinction between perceptions and demonstrated effects at the Results; Discussion, lines ~423–476), the results compellingly describe informants’ perceptions of BFM utility; however, in several sections, the language could more clearly distinguish perceived benefits from demonstrated or measured outcomes. Strengthening this distinction would reduce the risk of over-interpretation and maintain analytical precision**.** | **Response to Reviewers #1 and #2 (regarding distinction between perceptions and demonstrated effects):**  We thank the reviewers for highlighting the importance of clearly distinguishing stakeholder perceptions from demonstrated or measured program outcomes.  As this study was designed as an exploratory qualitative inquiry, our findings reflect informant perspectives on the perceived utility and operational pathways of BFM rather than empirically measured effects on drug coverage, equity, or sustainability.  In response, we have made several revisions throughout the manuscript to strengthen this distinction. Firstly, we added a clarifying statement at the end of the first paragraph of the Discussion explicitly noting that findings should be interpreted as describing perceived mechanisms and potential pathways rather than demonstrated causal effects.  Second, we have tempered language in the Abstract, Results, Discussion, and Conclusions to ensure that statements implying effectiveness are clearly framed as stakeholder perceptions or areas requiring further empirical evaluation. Finally, we strengthened the Limitations section to more explicitly acknowledge that findings reflect stakeholder perspectives rather than direct community experiences or measured program outcomes.  We have carefully reviewed and revised the manuscript to ensure this distinction is applied consistently throughout and are happy to make any further refinements if needed. |
| Based on the balance between ASCEND-specific and generalizable findings particularly at the results and discussion, Given the centrality of ASCEND examples, the authors should consider explicitly separating ASCEND-specific operational insights from those likely applicable across NTD programs. This would help readers understand which findings are context-bound versus broadly informative. | Thank you for this suggestion.  We agree that clarifying the distinction between ASCEND-specific operational mechanisms and more broadly applicable insights strengthens interpretability. We have revised the opening paragraph of the Discussion to explicitly frame ASCEND as an illustrative implementation case conducted within WHO recommended preventive chemotherapy frameworks.  This clarifies that while certain operational examples draw from ASCEND supported efforts to formalize BFM, the broader lessons identified relate to structural features common to preventive chemotherapy NTD programs.  We have furthermore made efforts throughout the manuscript to add precision when discussing transferability, including clarifying contextual boundaries in the Methods and Strengths and Limitations sections. |
| **Discussion** |  |
| - Integration of community-level perspectives at the Limitations section (lines 516–518) The limitation regarding absence of direct community voices is appropriately acknowledged. The manuscript could be strengthened by briefly discussing how future research might integrate community perspectives alongside key informant insights, particularly to validate or challenge professional interpretations of BFM effectiveness. | Thank you for this helpful suggestion. We agree that incorporating direct community perspectives is an important next step in strengthening the evidence base for BFM in MDA programs.  We have expanded the Recommendations section to briefly outline how future research could integrate community voices (such as through focus group discussions, participatory workshops, or mixed-method designs) to validate or challenge professional interpretations of BFM effectiveness. We believe this addition strengthens the forward-looking relevance of the manuscript while clarifying the scope of the current study. |
| Presentation and synthesis of Table 2 (Table 2; Discussion, lines ~419–428) Table 2 effectively summarizes findings and recommendations; however, the **authors should consider more explicitly linking each recommendation back to specific empirical findings in the Results section**, either through cross-referencing or clearer narrative signposting. | Thank you for this helpful suggestion.  We agree that strengthening the linkage between empirical findings and the recommendations presented in Table 2 improves clarity and analytical transparency. In response, we have revised the opening paragraph of the Discussion to explicitly state that Table 2 synthesizes themes identified in the Results section and presents operational recommendations derived directly from these findings.  We have also incorporated clearer narrative signposting throughout the Discussion to link key recommendations back to the corresponding thematic findings (like improving coverage and reaching missed populations; structural challenges to BFM integration; sustaining trust and closing the loop, now all cross referenced with Table 2). |
| looking at the recommendations and feasibility considerations (lines ~519–536) The recommendations are well-aligned with findings, but **feasibility considerations such as resource constraints, workforce capacity, and competing program priorities could be briefly discussed to contextualize implementation realism**, especially in lower-capacity settings. | Thank you for this important observation.    We agree that acknowledging implementation realities strengthens the practical relevance of the recommendations. In response, we have added a brief feasibility consideration within the Recommendations section that explicitly recognizes resource constraints, workforce capacity limitations, and competing program priorities in many endemic settings. We also note that phased integration leveraging existing tools and M&E personnel may enhance feasibility, particularly in lower-capacity contexts. |
| Language precision and consistency throughout manuscript is noted and it is **important that minor improvements should be made by ensuring consistent use of terminology (e.g., BFM vs. community feedback vs. participatory feedback)** and **avoiding occasional normative phrasing that may imply endorsement rather than analysis.** This would further enhance analytical neutrality. | Thank you for this helpful comment.  We have carefully reviewed the manuscript to ensure consistent use of terminology throughout. Where appropriate, we have standardized references to “beneficiary feedback mechanisms (BFM)” when referring specifically to the structured mechanisms examined in this study, while retaining broader terms such as “community feedback” only when referencing wider literature or general participatory approaches.  In addition, we have revised selected passages to reduce normative phrasing. We have also added a clarifying sentence in the Introduction defining how the term BFM is used in this manuscript to improve conceptual precision. We believe these revisions enhance clarity and consistency across the text. |
| The authors could strengthen policy relevance by **more clearly articulating how their findings could inform specific elements of guideline development** (e.g., indicators, minimum standards, accountability loops). | Thank you for this suggestion.  We have revised the final paragraph of the Recommendations section to more explicitly articulate how the findings could inform guideline development. Specifically, we now recommend integration of standardized BFM components into preventive chemotherapy distribution guidance, coverage evaluation survey guidelines, and broader M&E frameworks.  We also clarify that future empirical studies should inform the development of standardized BFM questions, reporting metrics, and practical guidance on effectively closing the feedback loop with communities. Given the current evidence base, we have intentionally avoided prescribing specific indicators, and instead emphasize the need for empirically informed standardization. |

**Reviewer #3**

| **General Comments** |  |
| --- | --- |
| This is a useful and insightful paper, highly relevant to NTD control and elimination efforts at both global and national levels. It has strong potential to improve program coverage, quality, accountability, and community ownership through continuous engagement. The paper is clearly aligned with the WHO NTD 2030 Roadmap, particularly for last-mile programs implementing MDAs, including those in sub-Saharan Africa. The lessons presented will be of immense value to other programs. I therefore recommend this paper for publication without any modifications. | Thank you for this feedback. |
| **Methods** |  |
| The objective of this paper is clearly stated. It indicates this study aims to explore how beneficiary feedback mechanisms (BFM) can be effectively operationalized to improve mass drug administration (MDA) programs for neglected tropical diseases (NTDs), with a specific focus on community feedback related to MDA implementation. The background information is adequate and provides sufficient justification for the study. The objectives are clearly stated and logically flow from the contextual background, establishing the relevance of the work to community-based programs and mass drug administration (MDA) efforts. For a qualitative study, the use of convenience sampling is acceptable and justified. | Thank you for this feedback. |
| **However, the absence of focus group discussions, which would have required additional fieldwork, represents a missed opportunity to deepen insights.** While such discussions would have deviated from the questionnaire-based tools applied**, their inclusion could have added greater richness to the findings and strengthened the recommendations**.  The study relied on semi-structured key informant interviews, without incorporating focus group discussions or broader field-level engagement. **The non-involvement of community members who form the foundation of community-led and community-based programs is a notable limitation.** That said, studies are expected to select methods most suitable for addressing their primary research questions, and the approach adopted here appropriately identifies areas that could be explored in subsequent research. | Thank you for this helpful suggestion. We agree that incorporating direct community perspectives is an important next step in strengthening the evidence base for BFM in MDA programs. We have expanded the Recommendations section to briefly outline how future research could integrate community voices—such as through focus group discussions, participatory workshops, or mixed-method designs—to validate, refine, or challenge professional interpretations of BFM effectiveness. We believe this addition strengthens the forward-looking relevance of the manuscript while clarifying the scope of the current study. |
| **Results** |  |
| The qualitative analysis undertaken is acceptable, and the results are clearly presented. The findings are coherent and well aligned with the study objectives, particularly in table 2, which summarizes the key findings and associated recommendations. It is particularly impressive. The alignment between findings and recommendations is clear and strengthens the practical value of the paper. | Thank you for this feedback. |
| The limitations of the study are noted in the paper; however, the paper would have benefited **from greater emphasis on health systems strengthening beyond MDAs, NTDs, and other infectious diseases, especially programs that rely heavily on sustained community engagement.** | Thank you for this comment.  We agree that structured feedback mechanisms have relevance beyond NTD and MDA programming, particularly in health system strengthening initiatives that depend on sustained community engagement. While a detailed exploration of these broader applications was beyond the scope of the present study, we have acknowledged this in the Discussion as an important area for future research. |
| Additionally, **the discussion on program sustainability could have been slightly expanded to enhance the overall utility of the paper. While** the paper underscores the importance of community engagement, social mobilization, and drug coverage validation**, it stops short of prescribing concrete methods for systematically collecting, analyzing, and responding to beneficiary feedback in the study.** Addressing this gap would have strengthened the operational relevance of the study. | Thank you for this constructive comment.  We agree that articulating the operational implications of beneficiary feedback mechanisms (BFM) is important for enhancing the practical utility of the study.  In response, we have strengthened the Abstract, Background, and Discussion to more explicitly highlight the study’s contribution to program sustainability and operational learning. In particular, we clarify that this manuscript differs from prior implementation reports or toolkits by providing cross-stakeholder analysis of how BFM are perceived, integrated, and operationalized within routine MDA programming, rather than evaluating a single predefined model.  At the same time, we deliberately avoided prescribing specific mechanisms for systematically collecting, analyzing, and responding to beneficiary feedback. Our qualitative design did not aim to compare models of BFM implementation or determine which approaches are most effective in improving drug coverage or equity. Given the heterogeneity of program contexts and delivery platforms, we believe that prescriptive recommendations without comparative empirical evaluation risk oversimplification.  We have therefore revised the Discussion to emphasize that a critical next step is prospective evaluation of different BFM models, including structured trials or implementation research assessing their feasibility, cost, and impact on key MDA indicators such as coverage, equity, adherence, and community trust. We agree that strengthening this evidence base will be essential to advancing operational guidance for NTD programs. |
| **Conclusions** |  |
| The objective of this paper is clear and offers valuable lessons for other programs regarding both the successes and challenges of beneficiary feedback mechanisms (BFM). The study effectively highlights the strategic role of BFM in reaching systematically missed populations and improving program responsiveness. In other words, the conclusions are supported by the data presented. | Thank you for this feedback. |
