# Supplementary material for: Beneficiary feedback mechanisms: Exploring ways to systematically integrate community feedback in mass drug administration delivery for NTDs
Source: PLoS Negl Trop Dis. 2026 Jun 8;20(6):e0014373. doi: 10.1371/journal.pntd.0014373 (PMC13258146; doi:10.1371/journal.pntd.0014373)
Supplement: Attachment — Submitted filename: PNTD-D-25-02037_R1_Reviewer_Feedback.docx [file pntd.0014373.s004.docx]

**General Comment**

I appreciate the authors for addressing a topic of significant scientific importance.
The manuscript titled " ***Beneficiary Feedback Mechanisms: Exploring ways to systematically integrate community feedback in mass drug administration delivery for NTDs***" is highly relevant, particularly for communities on the last mile of NTD elimination as targeted on the WHO 2021-2030 NTD Roadmap.

**Background**

The authors present essential evidence on the current state of the art regarding the topic. Reading the comments from previous Reviewers 1, 2 and 3, I must commend the authors for addressing the comments made by previous reviewers, which I believe would have helped in improving the current reviewed version.

**Methods**

The authors have outlined a well-structured methodology, which reads very clearly. In this updated version of the manuscript, the authors have addressed questions from previous reviewers with respect to participants recruitment process, the thematic analysis approach applied which details. These added details made the manuscript better in understanding by different audiences and stakeholders as well as the larger scientific community.

**Results**

On the different topic-related sections reported, the authors used quotes that evidently include the participants` direct voices on the manuscript, which is very essential for a qualitative study with 14 participants. Most especially, including the voices of stakeholders from different levels of NTD program implementation enriches the results herein. The authors present participants’ original opinions, which allows readers to engage with their perspectives directly, rather than rely only on the author’s interpretation.

**Discussion and Conclusion**

The authors have effectively discussed the contribution of their work to the importance of integrating Beneficiary Feedback Mechanisms in mass drug administration delivery for NTDs. They have clearly outlined the strengths and limitations of the study. Most importantly, the authors have made very relevant recommendations that would enable better implementation of the BFM by national NTD control programmes.

**References**

Line 616: The article title is written in italics, which is different from other titles.

Line 643: The article is written with each word capitalized, which is different from other titles.

Line 692, 698, 701,704, 725, 732, 741, 746, 756, 767 & 781: See previous comment

**Final decision**

This revised version of the manuscript title “***Beneficiary Feedback Mechanisms: Exploring ways to systematically integrate community feedback in mass drug administration delivery for NTDs”*** is highly essential to inform NTD control programs, researchers and policymakers working on NTDs. After effecting the minor corrections herein, I recommend this manuscript for publication by PLOS NTDs.
