# Supplementary material for: Beneficiary feedback mechanisms: Exploring ways to systematically integrate community feedback in mass drug administration delivery for NTDs
Source: PLoS Negl Trop Dis. 2026 Jun 8;20(6):e0014373. doi: 10.1371/journal.pntd.0014373 (PMC13258146; doi:10.1371/journal.pntd.0014373)
Supplement: Attachment — Submitted filename: Response to Reviewers.docx [file pntd.0014373.s006.docx]

Dear reviewers,

Thank you for your careful review of our manuscript and for the positive feedback. We greatly appreciate your comments and support for this work.

In response to the formatting issues identified in the reference list, we conducted a careful review and revision of all citations. We corrected inconsistencies in capitalization and italicization, standardized formatting throughout the reference list, removed duplicate entries, and updated the citation style to remove unwanted metadata associated with certain references. Please find these updates reflected in the revised manuscript.

Finally, we took this opportunity to conduct a thorough copy edit, making very minor grammatical and formatting corrections without changing the content of the manuscript.

Thank you again for your time, constructive feedback, and support throughout the review process.

Sincerely,
Jake Mathewson and co-authors
