# Supplementary material for: Beneficiary feedback mechanisms: Exploring ways to systematically integrate community feedback in mass drug administration delivery for NTDs
Source: PLoS Negl Trop Dis. 2026 Jun 8;20(6):e0014373. doi: 10.1371/journal.pntd.0014373 (PMC13258146; doi:10.1371/journal.pntd.0014373)
Supplement: Peer review history

# Peer review: Decision Letter 0

**DOI:** 10.1371/journal.pntd.0014373.r001

**Authors:** Robert Adamu SHEY

11 Feb 2026

PNTD-D-25-02037

Beneficiary Feedback Mechanisms: Exploring ways to systematically integrate community feedback in mass drug administration delivery for NTDs

PLOS Neglected Tropical Diseases

Dear Dr. Mathewson,

Thank you for submitting your manuscript to PLOS Neglected Tropical Diseases. After careful consideration, we feel that it has merit but does not fully meet PLOS Neglected Tropical Diseases's publication criteria as it currently stands. Therefore, we invite you to submit a revised version of the manuscript that addresses the points raised during the review process.

Please submit your revised manuscript within by Apr 12 2026 11:59PM. If you will need more time than this to complete your revisions, please reply to this message or contact the journal office at plosntds@plos.org. When you're ready to submit your revision, log on to https://www.editorialmanager.com/pntd/ and select the 'Submissions Needing Revision' folder to locate your manuscript file.

Please include the following items when submitting your revised manuscript:

* A letter that responds to each point raised by the editor and reviewer(s). You should upload this letter as a separate file labeled 'Response to Reviewers'. This file does not need to include responses to any formatting updates and technical items listed in the 'Journal Requirements' section below.

* A marked-up copy of your manuscript that highlights changes made to the original version. You should upload this as a separate file labeled 'Revised Manuscript with Track Changes'.

* An unmarked version of your revised paper without tracked changes. You should upload this as a separate file labeled 'Manuscript'.

If you would like to make changes to your financial disclosure, competing interests statement, or data availability statement, please make these updates within the submission form at the time of resubmission. Guidelines for resubmitting your figure files are available below the reviewer comments at the end of this letter.

We look forward to receiving your revised manuscript.

Kind regards,

Robert Adamu SHEY, Ph.D.

Guest Editor

PLOS Neglected Tropical Diseases

Jong-Yil Chai

Section Editor

PLOS Neglected Tropical Diseases

Shaden Kamhawi

co-Editor-in-Chief

PLOS Neglected Tropical Diseases

orcid.org/0000-0003-4304-636XX

Paul Brindley

co-Editor-in-Chief

PLOS Neglected Tropical Diseases

orcid.org/0000-0003-1765-0002

**Journal Requirements:**

1) <carina-action-element class="ng-star-inserted">Please ensure that the CRediT author contributions listed for every co-author are completed accurately and in full.</carina-action-element>

--><carina-action-element class="ng-star-inserted">At this stage, the following Authors/Authors require contributions: </carina-action-element><carina-action-element class="ng-star-inserted">Jake D. Mathewson, Charlie Aardewijn, Sake J de Vlas, Dunstan J Matungwa, and Mirjam I Bakker</carina-action-element><carina-action-element class="ng-star-inserted">. Please ensure that the full contributions of each author are acknowledged in the "Add/Edit/Remove Authors" section of our submission form.</carina-action-element>

<carina-action-element class="ng-star-inserted">The list of CRediT author contributions may be found here: https://journals.plos.org/</carina-action-element><carina-action-element class="ng-star-inserted">plosntds</carina-action-element><carina-action-element class="ng-star-inserted">/s/authorship#loc-author-contributions</carina-action-element>

2) <carina-action-element class="ng-star-inserted">Please provide an Author Summary. This should appear in your manuscript between the Abstract (if applicable) and the Introduction, and should be 150-200 words long. The aim should be to make your findings accessible to a wide audience that includes both scientists and non-scientists. Sample summaries can be found on our website under Submission Guidelines:</carina-action-element>

--><carina-action-element class="ng-star-inserted">https://journals.plos.org/</carina-action-element><carina-action-element class="ng-star-inserted">plosntds</carina-action-element><carina-action-element class="ng-star-inserted">/s/submission-guidelines#loc-parts-of-a-submission</carina-action-element>

3) <carina-action-element class="ng-star-inserted">Please upload all main figures as separate Figure files in .tif or .eps format. For more information about how to convert and format your figure files please see our guidelines: </carina-action-element>

--><carina-action-element class="ng-star-inserted">https://journals.plos.org/</carina-action-element><carina-action-element class="ng-star-inserted">plosntds</carina-action-element><carina-action-element class="ng-star-inserted">/s/figures</carina-action-element>

4) <carina-action-element class="ng-star-inserted">Some material included in your submission may be copyrighted. According to PLOSu2019s copyright policy, authors who use figures or other material (e.g., graphics, clipart, maps) from another author or copyright holder must demonstrate or obtain permission to publish this material under the Creative Commons Attribution 4.0 International (CC BY 4.0) License used by PLOS journals. Please closely review the details of PLOSu2019s copyright requirements here: PLOS Licenses and Copyright. If you need to request permissions from a copyright holder, you may use PLOS's Copyright Content Permission form.</carina-action-element>

--><carina-action-element class="ng-star-inserted">Please respond directly to this email and provide any known details concerning your material's license terms and permissions required for reuse, even if you have not yet obtained copyright permissions or are unsure of your material's copyright compatibility. Once you have responded and addressed all other outstanding technical requirements, you may resubmit your manuscript within Editorial Manager. </carina-action-element>

<carina-action-element class="ng-star-inserted">Potential Copyright Issues:</carina-action-element>

<carina-action-element class="ng-star-inserted">i) Figure 1.. Please confirm whether you drew the images / clip-art within the figure panels by hand. If you did not draw the images, please provide (a) a link to the source of the images or icons and their license / terms of use; or (b) written permission from the copyright holder to publish the images or icons under our CC BY 4.0 license. Alternatively, you may replace the images with open source alternatives. See these open source resources you may use to replace images / clip-art: - https://commons.wikimedia.org - https://openclipart.org/. </carina-action-element>

**Reviewers' Comments:**

Reviewer's Responses to Questions

**Key Review Criteria Required for Acceptance?**

As you describe the new analyses required for acceptance, please consider the following:

**Methods**

-Are the objectives of the study clearly articulated with a clear testable hypothesis stated?

-Is the study design appropriate to address the stated objectives?

-Is the population clearly described and appropriate for the hypothesis being tested?

-Is the sample size sufficient to ensure adequate power to address the hypothesis being tested?

-Were correct statistical analysis used to support conclusions?

-Are there concerns about ethical or regulatory requirements being met?

Reviewer #1: The objectives are clearly stated and appropriate for an implementation-focused qualitative study. A qualitative, key-informant interview design is appropriate for exploring stakeholder perspectives on beneficiary feedback mechanisms in MDA delivery. The study population (implementers, donors, and technical partners) is clearly described and relevant to the objectives. However, findings reflect stakeholder perceptions rather than direct community or beneficiary experiences, which should be more clearly emphasized. For qualitative research, the sample size (14 informants) is acceptable, but the manuscript would benefit from clearer justification regarding saturation and representation across stakeholder groups.

No statistical analysis was presented as this is a qualitative study. However, additional detail is needed on qualitative analytic procedures, including how transcripts were sampled for double coding and how coding disagreements were resolved. No concerns for ethical/regulatory requirements.

Reviewer #2: (No Response)

Reviewer #3: The objective of this paper is clearly stated. It indicates this study aims to explore how beneficiary feedback mechanisms (BFM) can be effectively operationalized to improve mass drug administration (MDA) programs for neglected tropical diseases (NTDs), with a specific focus on community feedback related to MDA implementation.

The background information is adequate and provides sufficient justification for the study. The objectives are clearly stated and logically flow from the contextual background, establishing the relevance of the work to community-based programs and mass drug administration (MDA) efforts.

For a qualitative study, the use of convenience sampling is acceptable and justified. However, the absence of focus group discussions, which would have required additional fieldwork, represents a missed opportunity to deepen insights. While such discussions would have deviated from the questionnaire-based tools applied, their inclusion could have added greater richness to the findings and strengthened the recommendations.

The study relied on semi-structured key informant interviews, without incorporating focus group discussions or broader field-level engagement. The non-involvement of community members who form the foundation of community-led and community-based programs is a notable limitation. That said, studies are expected to select methods most suitable for addressing their primary research questions, and the approach adopted here appropriately identifies areas that could be explored in subsequent research.

**********

**Results**

-Does the analysis presented match the analysis plan?

-Are the results clearly and completely presented?

-Are the figures (Tables, Images) of sufficient quality for clarity?

Reviewer #1: The analysis presented is generally consistent with the stated qualitative analytic approach, but additional detail on the execution of the analysis plan (e.g. sampling for double coding and resolution of coding discrepancies) would be helpful, to improve transparency. The results are clearly written and logically organized, with key themes presented in a coherent manner, although some conclusions would benefit from clearer separation between perceived mechanisms and demonstrated outcomes. The tables and figures are of generally good quality and support the text.

Reviewer #2: (No Response)

Reviewer #3: The qualitative analysis undertaken is acceptable, and the results are clearly presented. The findings are coherent and well aligned with the study objectives, particularly in table 2, which summarizes the key findings and associated recommendations. It is particularly impressive. The alignment between findings and recommendations is clear and strengthens the practical value of the paper.

The limitations of the study are noted in the paper; however, the paper would have benefited from greater emphasis on health systems strengthening beyond MDAs, NTDs, and other infectious diseases, especially programs that rely heavily on sustained community engagement. Additionally, the discussion on program sustainability could have been slightly expanded to enhance the overall utility of the paper.

While the paper underscores the importance of community engagement, social mobilization, and drug coverage validation, it stops short of prescribing concrete methods for systematically collecting, analyzing, and responding to beneficiary feedback in the study. Addressing this gap would have strengthened the operational relevance of the study.

**********

**Conclusions**

-Are the conclusions supported by the data presented?

-Are the limitations of analysis clearly described?

-Do the authors discuss how these data can be helpful to advance our understanding of the topic under study?

-Is public health relevance addressed?

Reviewer #1: Overall, the conclusions are generally supported by the qualitative data presented, particularly with respect to stakeholder perceptions of the value and feasibility of beneficiary feedback mechanisms in MDA delivery. However, some conclusions—especially those implying improvements in coverage or equity—would benefit from clearer qualification to distinguish perceived pathways from empirically demonstrated effects. The authors do acknowledge several key limitations, including potential selection bias and the lack of direct beneficiary perspectives, though these limitations could be more prominently emphasized. The manuscript clearly discusses how the findings can inform implementation practice and program design, and the public health relevance is well articulated, given the importance of community engagement for effective and equitable NTD control and elimination programs.

Reviewer #2: (No Response)

Reviewer #3: The objective of this paper is clear and offers valuable lessons for other programs regarding both the successes and challenges of beneficiary feedback mechanisms (BFM). The study effectively highlights the strategic role of BFM in reaching systematically missed populations and improving program responsiveness. In other words, the conclusions are supported by the data presented.

**********

**Editorial and Data Presentation Modifications?**

Use this section for editorial suggestions as well as relatively minor modifications of existing data that would enhance clarity. If the only modifications needed are minor and/or editorial, you may wish to recommend “Minor Revision” or “Accept”.

Reviewer #1: (No Response)

Reviewer #2: (No Response)

Reviewer #3: No revisions required.

**********

**Summary and General Comments**

Use this section to provide overall comments, discuss strengths/weaknesses of the study, novelty, significance, general execution and scholarship. You may also include additional comments for the author, including concerns about dual publication, research ethics, or publication ethics. If requesting major revision, please articulate the new experiments that are needed.

Reviewer #1: This is a timely and relevant implementation-focused qualitative study addressing an important operational gap in NTD programs: the systematic collection and integration of community feedback to improve MDA delivery. The manuscript is clearly written, based on a well-designed interview approach, and presents plausible and programmatically relevant findings. Its main strengths lie in its focus on an underexplored aspect of MDA implementation and its synthesis of stakeholder perspectives across multiple roles, contributing useful operational insights for NTD programs.

However, the manuscript’s novelty is primarily conceptual rather than empirical, and several methodological and interpretive issues require attention. Greater transparency is needed regarding qualitative analytic procedures, including what constituted the analytic sample for double coding and how coding disagreements were resolved. Claims of “external validity” appear overstated given the small, network-based sample largely drawn from ASCEND-supported contexts; the findings are better framed as transferable rather than externally validated. In addition, some statements imply effectiveness (e.g., improved coverage or equity) based on anecdotal examples and should be tempered to clearly distinguish perceived pathways from demonstrated outcomes. Finally, clearer specification of minimal or essential components of beneficiary feedback mechanisms within MDA would strengthen the paper’s practical contribution.

Reviewer #2: (No Response)

Reviewer #3: This is a useful and insightful paper, highly relevant to NTD control and elimination efforts at both global and national levels. It has strong potential to improve program coverage, quality, accountability, and community ownership through continuous engagement. The paper is clearly aligned with the WHO NTD 2030 Roadmap, particularly for last-mile programs implementing MDAs, including those in sub-Saharan Africa.

The lessons presented will be of immense value to other programs. I therefore recommend this paper for publication without any modifications.

**********

PLOS authors have the option to publish the peer review history of their article (what does this mean?). If published, this will include your full peer review and any attached files.

If you choose “no”, your identity will remain anonymous but your review may still be made public.

**Do you want your identity to be public for this peer review?**  For information about this choice, including consent withdrawal, please see our Privacy Policy.

Reviewer #1: No

Reviewer #2: No

Reviewer #3: **Yes:** Nana Kwadwo Biritwum

[NOTE: If reviewer comments were submitted as an attachment file, they will be attached to this email and accessible via the submission site. Please log into your account, locate the manuscript record, and check for the action link "View Attachments". If this link does not appear, there are no attachment files.]

**Figure resubmission:**

-->While revising your submission, we strongly recommend that you use PLOS’s NAAS tool (https://ngplosjournals.pagemajik.ai/artanalysis) to test your figure files. NAAS can convert your figure files to the TIFF file type and meet basic requirements (such as print size, resolution), or provide you with a report on issues that do not meet our requirements and that NAAS cannot fix.

After uploading your figures to PLOS’s NAAS tool - https://ngplosjournals.pagemajik.ai/artanalysis, NAAS will process the files provided and display the results in the "Uploaded Files" section of the page as the processing is complete. If the uploaded figures meet our requirements (or NAAS is able to fix the files to meet our requirements), the figure will be marked as "fixed" above. If NAAS is unable to fix the files, a red "failed" label will appear above. When NAAS has confirmed that the figure files meet our requirements, please download the file via the download option, and include these NAAS processed figure files when submitting your revised manuscript.

**Reproducibility:**

To enhance the reproducibility of your results, we recommend that authors of applicable studies deposit laboratory protocols in protocols.io, where a protocol can be assigned its own identifier (DOI) such that it can be cited independently in the future. Additionally, PLOS ONE offers an option to publish peer-reviewed clinical study protocols. Read more information on sharing protocols at https://plos.org/protocols?utm_medium=editorial-email&utm_source=authorletters&utm_campaign=protocols

Attachment Submitted filename: Reviewers FeedBack.docx

# Author comment: Author response to Decision Letter 1

**DOI:** 10.1371/journal.pntd.0014373.r002

27 Feb 2026

Attachment Submitted filename: BFM Response to Reviewers_24Feb.docx

# Peer review: Decision Letter 1

**DOI:** 10.1371/journal.pntd.0014373.r003

**Authors:** Robert Adamu SHEY

6 May 2026

PNTD-D-25-02037R1

Beneficiary Feedback Mechanisms: Exploring ways to systematically integrate community feedback in mass drug administration delivery for NTDs

PLOS Neglected Tropical Diseases

Dear Dr. Mathewson,

Thank you for submitting your manuscript to PLOS Neglected Tropical Diseases. After careful consideration, we feel that it has merit but does not fully meet PLOS Neglected Tropical Diseases's publication criteria as it currently stands. Therefore, we invite you to submit a revised version of the manuscript that addresses the points raised during the review process.

Please submit your revised manuscript by Jun 05 2026 11:59PM. If you will need more time than this to complete your revisions, please reply to this message or contact the journal office at plosntds@plos.org. When you're ready to submit your revision, log on to https://www.editorialmanager.com/pntd/ and select the 'Submissions Needing Revision' folder to locate your manuscript file.

Please include the following items when submitting your revised manuscript:

* A letter that responds to each point raised by the editor and reviewer(s). You should upload this letter as a separate file labeled 'Response to Reviewers'. This file does not need to include responses to any formatting updates and technical items listed in the 'Journal Requirements' section below.

* A marked-up copy of your manuscript that highlights changes made to the original version. You should upload this as a separate file labeled 'Revised Manuscript with Track Changes'.

* An unmarked version of your revised paper without tracked changes. You should upload this as a separate file labeled 'Manuscript'.

If you would like to make changes to your financial disclosure, competing interests statement, or data availability statement, please make these updates within the submission form at the time of resubmission. Guidelines for resubmitting your figure files are available below the reviewer comments at the end of this letter.

As the corresponding author, your ORCID iD is verified in the submission system and will appear in the published article. PLOS supports the use of ORCID, and we encourage all coauthors to register for an ORCID iD and use it as well. Please encourage your coauthors to verify their ORCID iD within the submission system before final acceptance, as unverified ORCID iDs will not appear in the published article. Only the individual author can complete the verification step; PLOS staff cannot verify ORCID iDs on behalf of authors.

We look forward to receiving your revised manuscript.

Kind regards,

Robert Adamu SHEY, Ph.D.

Guest Editor

PLOS Neglected Tropical Diseases

Jong-Yil Chai

Section Editor

PLOS Neglected Tropical Diseases

Shaden Kamhawi

co-Editor-in-Chief

PLOS Neglected Tropical Diseases

orcid.org/0000-0003-4304-636XX

Paul Brindley

co-Editor-in-Chief

PLOS Neglected Tropical Diseases

orcid.org/0000-0003-1765-0002

**Journal Requirements:**

Please provide an Author Summary. This should appear in your manuscript between the Abstract (if applicable) and the Introduction, and should be 150-200 words long. The aim should be to make your findings accessible to a wide audience that includes both scientists and non-scientists. Sample summaries can be found on our website under Submission Guidelines:

https://journals.plos.org/plosntds/s/submission-guidelines#loc-parts-of-a-submission

**Reviewers' comments:**

Reviewer's Responses to Questions

**Key Review Criteria Required for Acceptance?**

As you describe the new analyses required for acceptance, please consider the following:

**Methods**

-Are the objectives of the study clearly articulated with a clear testable hypothesis stated?

-Is the study design appropriate to address the stated objectives?

-Is the population clearly described and appropriate for the hypothesis being tested?

-Is the sample size sufficient to ensure adequate power to address the hypothesis being tested?

-Were correct statistical analysis used to support conclusions?

-Are there concerns about ethical or regulatory requirements being met?

Reviewer #4: Key review criteria were met in this manuscript.

Reviewer #5: The authors have outlined a well-structured methodology, which reads very clearly. In this updated version of the manuscript, the authors have addressed questions from previous reviewers with respect to participants recruitment process, the thematic analysis approach applied which details. These added details made the manuscript better in understanding by different audiences and stakeholders as well as the larger scientific community.

Reviewer #6: Yes to objectives, study design, description of population. Sample size and statistical analysis not applicable. No ethical concerns

**********

**Results**

-Does the analysis presented match the analysis plan?

-Are the results clearly and completely presented?

-Are the figures (Tables, Images) of sufficient quality for clarity?

Reviewer #4: key criteria for "results" description was also met.

Reviewer #5: On the different topic-related sections reported, the authors used quotes that evidently include the participants` direct voices on the manuscript, which is very essential for a qualitative study with 14 participants. Most especially, including the voices of stakeholders from different levels of NTD program implementation enriches the results herein. The authors present participants’ original opinions, which allows readers to engage with their perspectives directly, rather than rely only on the author’s interpretation.

Reviewer #6: Yes to all

**********

**Conclusions**

-Are the conclusions supported by the data presented?

-Are the limitations of analysis clearly described?

-Do the authors discuss how these data can be helpful to advance our understanding of the topic under study?

-Is public health relevance addressed?

Reviewer #4: Key criteria for "conclusions" were also met.

Reviewer #5: The authors have effectively discussed the contribution of their work to the importance of integrating Beneficiary Feedback Mechanisms in mass drug administration delivery for NTDs. They have clearly outlined the strengths and limitations of the study. Most importantly, the authors have made very relevant recommendations that would enable better implementation of the BFM by national NTD control programmes.

Reviewer #6: Yes to all

**********

**Editorial and Data Presentation Modifications?**

Use this section for editorial suggestions as well as relatively minor modifications of existing data that would enhance clarity. If the only modifications needed are minor and/or editorial, you may wish to recommend “Minor Revision” or “Accept”.

Reviewer #4: Accept as it is now, after R1 review.

Reviewer #5: (No Response)

Reviewer #6: The paper is clearly written and no modifications are needed

**********

**Summary and General Comments**

Use this section to provide overall comments, discuss strengths/weaknesses of the study, novelty, significance, general execution and scholarship. You may also include additional comments for the author, including concerns about dual publication, research ethics, or publication ethics. If requesting major revision, please articulate the new experiments that are needed.

Reviewer #4: The manuscript delivers a well designed study and well written report. Authors have adequately answered R1 reviewers comments and suggestions.

Reviewer #5: General Comment

I appreciate the authors for addressing a topic of significant scientific importance.

The manuscript titled " Beneficiary Feedback Mechanisms: Exploring ways to systematically integrate community feedback in mass drug administration delivery for NTDs" is highly relevant, particularly for communities on the last mile of NTD elimination as targeted on the WHO 2021-2030 NTD Roadmap.

Final decision

This revised version of the manuscript title “Beneficiary Feedback Mechanisms: Exploring ways to systematically integrate community feedback in mass drug administration delivery for NTDs” is highly essential to inform NTD control programs, researchers and policymakers working on NTDs. After effecting the minor corrections herein, I recommend this manuscript for publication by PLOS NTDs.

Reviewer #6: This is an important paper describing the importance of community feedback in ensuring good coverage for MDA programmes for NTDs, discussing the best ways to collect this feedback and use it to engage communities. The minor weaknesses and possible biases in the study are clearly stated and the recommendations are clear.

**********

PLOS authors have the option to publish the peer review history of their article (what does this mean?). If published, this will include your full peer review and any attached files.

If you choose “no”, your identity will remain anonymous but your review may still be made public.

**Do you want your identity to be public for this peer review?**  For information about this choice, including consent withdrawal, please see our Privacy Policy.

Reviewer #4: **Yes:** Carlos Graeff-Teixeira

Reviewer #5: No

Reviewer #6: **Yes:** David Mabey

[NOTE: If reviewer comments were submitted as an attachment file, they will be attached to this email and accessible via the submission site. Please log into your account, locate the manuscript record, and check for the action link "View Attachments". If this link does not appear, there are no attachment files.]

**Figure resubmission:**

While revising your submission, we strongly recommend that you use PLOS’s NAAS tool (https://ngplosjournals.pagemajik.ai/artanalysis) to test your figure files. NAAS can convert your figure files to the TIFF file type and meet basic requirements (such as print size, resolution), or provide you with a report on issues that do not meet our requirements and that NAAS cannot fix.

After uploading your figures to PLOS’s NAAS tool - https://ngplosjournals.pagemajik.ai/artanalysis, NAAS will process the files provided and display the results in the "Uploaded Files" section of the page as the processing is complete. If the uploaded figures meet our requirements (or NAAS is able to fix the files to meet our requirements), the figure will be marked as "fixed" above. If NAAS is unable to fix the files, a red "failed" label will appear above. When NAAS has confirmed that the figure files meet our requirements, please download the file via the download option, and include these NAAS processed figure files when submitting your revised manuscript.

**Reproducibility:**

To enhance the reproducibility of your results, we recommend that authors of applicable studies deposit laboratory protocols in protocols.io, where a protocol can be assigned its own identifier (DOI) such that it can be cited independently in the future. Additionally, PLOS ONE offers an option to publish peer-reviewed clinical study protocols. Read more information on sharing protocols at https://plos.org/protocols?utm_medium=editorial-email&utm_source=authorletters&utm_campaign=protocols

Attachment Submitted filename: PNTD-D-25-02037_R1_Reviewer_Feedback.docx

# Author comment: Author response to Decision Letter 2

**DOI:** 10.1371/journal.pntd.0014373.r004

7 May 2026

Attachment Submitted filename: Response to Reviewers.docx

# Editor report: Decision Letter 2

**DOI:** 10.1371/journal.pntd.0014373.r005

**Authors:** Robert Adamu SHEY

12 May 2026

Dear Mr Mathewson,

We are pleased to inform you that your manuscript 'Beneficiary feedback mechanisms: exploring ways to systematically integrate community feedback in mass drug administration delivery for NTDs' has been provisionally accepted for publication in PLOS Neglected Tropical Diseases.

Before your manuscript can be formally accepted you will need to complete some formatting changes, which you will receive in a follow up email. A member of our team will be in touch with a set of requests.

Please note that your manuscript will not be scheduled for publication until you have made the required changes, so a swift response is appreciated.

IMPORTANT: The editorial review process is now complete. PLOS will only permit corrections to spelling, formatting or significant scientific errors from this point onwards. Requests for major changes, or any which affect the scientific understanding of your work, will cause delays to the publication date of your manuscript.

Should you, your institution's press office or the journal office choose to press release your paper, you will automatically be opted out of early publication. We ask that you notify us now if you or your institution is planning to press release the article. All press must be co-ordinated with PLOS.

Thank you again for supporting Open Access publishing; we are looking forward to publishing your work in PLOS Neglected Tropical Diseases.

Best regards,

Robert Adamu SHEY, Ph.D.

Guest Editor

PLOS Neglected Tropical Diseases

Jong-Yil Chai

Section Editor

PLOS Neglected Tropical Diseases

Shaden Kamhawi

co-Editor-in-Chief

PLOS Neglected Tropical Diseases

orcid.org/0000-0003-4304-636XX

Paul Brindley

co-Editor-in-Chief

PLOS Neglected Tropical Diseases

orcid.org/0000-0003-1765-0002

***********************************************************

p.p1 {margin: 0.0px 0.0px 0.0px 0.0px; line-height: 16.0px; font: 14.0px Arial; color: #323333; -webkit-text-stroke: #323333}span.s1 {font-kerning: none

# Editor report: Acceptance letter

**DOI:** 10.1371/journal.pntd.0014373.r006

**Authors:** Robert Adamu SHEY

Dear Mr Mathewson,

We are delighted to inform you that your manuscript, "Beneficiary feedback mechanisms: exploring ways to systematically integrate community feedback in mass drug administration delivery for NTDs," has been formally accepted for publication in PLOS Neglected Tropical Diseases.

We have now passed your article onto the PLOS Production Department who will complete the rest of the publication process. All authors will receive a confirmation email upon publication.

The corresponding author will soon be receiving a typeset proof for review, to ensure errors have not been introduced during production. Please review the PDF proof of your manuscript carefully, as this is the last chance to correct any scientific or type-setting errors. Please note that major changes, or those which affect the scientific understanding of the work, will likely cause delays to the publication date of your manuscript. Note: Proofs for Front Matter articles (Editorial, Viewpoint, Symposium, Review, etc...) are generated on a different schedule and may not be made available as quickly.

Soon after your final files are uploaded, the early version of your manuscript will be published online unless you opted out of this process. The date of the early version will be your article's publication date. The final article will be published to the same URL, and all versions of the paper will be accessible to readers.

For Research Articles, you will receive an invoice from PLOS for your publication fee after your manuscript has reached the completed accept phase. If you receive an email requesting payment before acceptance or for any other service, this may be a phishing scheme. Learn how to identify phishing emails and protect your accounts at https://explore.plos.org/phishing.

Thank you again for supporting open-access publishing; we are looking forward to publishing your work in PLOS Neglected Tropical Diseases.

Best regards,

Shaden Kamhawi

co-Editor-in-Chief

PLOS Neglected Tropical Diseases

Paul Brindley

co-Editor-in-Chief

PLOS Neglected Tropical Diseases
